# Variation in relapse frequency and the transmission potential of *Plasmodium vivax* malaria

**DOI:** 10.1098/rspb.2016.0048

**Published:** 2016-03-30

**Authors:** Michael T. White, George Shirreff, Stephan Karl, Azra C. Ghani, Ivo Mueller

**Affiliations:** 1 MRC Centre for Outbreak Analysis and Modelling, Department of Infectious Disease Epidemiology, Imperial College London, Norfolk Place, London W2 1PG, UK; 2 Division of Population Health and Immunity, Walter and Eliza Hall Institute, Melbourne, VIC 3052, Australia; 3 Department of Medical Biology, University of Melbourne, Melbourne, VIC 3010, Australia; 4 Centre de Recerca en Salut Internacional de Barcelona, 08036 Barcelona, Spain

**Keywords:** *Plasmodium vivax* malaria, relapse, mathematical model, transmission potential, seasonality

## Abstract

There is substantial variation in the relapse frequency of *Plasmodium vivax* malaria, with fast-relapsing strains in tropical areas, and slow-relapsing strains in temperate areas with seasonal transmission. We hypothesize that much of the phenotypic diversity in *P. vivax* relapses arises from selection of relapse frequency to optimize transmission potential in a given environment, in a process similar to the virulence trade-off hypothesis. We develop mathematical models of *P. vivax* transmission and calculate the basic reproduction number *R*_0_ to investigate how transmission potential varies with relapse frequency and seasonality. In tropical zones with year-round transmission, transmission potential is optimized at intermediate relapse frequencies of two to three months: slower-relapsing strains increase the opportunity for onward transmission to mosquitoes, but also increase the risk of being outcompeted by faster-relapsing strains. Seasonality is an important driver of relapse frequency for temperate strains, with the time to first relapse predicted to be six to nine months, coinciding with the duration between seasonal transmission peaks. We predict that there is a threshold degree of seasonality, below which fast-relapsing tropical strains are selected for, and above which slow-relapsing temperate strains dominate, providing an explanation for the observed global distribution of relapse phenotypes.

## 1. Introduction

*Plasmodium vivax* malaria is endemically transmitted or has been historically endemic across the globe in regions with extremely diverse climates [1,2], from temperate locations such as Finland [3] to Papua New Guinea in the tropics [4]. A notable exception is in large parts of sub-Saharan Africa where high prevalence of the Duffy-negative phenotype renders populations relatively resistant to *P. vivax* infection [5], even though the more virulent *Plasmodium falciparum* malaria is highly prevalent. A fundamental difference between these species of malaria is the ability of *P. vivax* parasites to remain latent in the liver following an infectious mosquito bite, activating weeks to months later to cause relapses [6]. Relapses play an important role in the transmission of *P. vivax* and account for its potential for sustained transmission in such a diverse range of environmental niches.

*Plasmodium vivax* is conventionally classified according to whether relapses follow a tropical or temperate phenotype [6–8]. In tropical regions with year-round mosquito-borne transmission, relapses occur rapidly at a frequency of three to six weeks. In temperate regions where mosquito-borne transmission is possible only in the summer months [1], the time to first relapse is typically 6–12 months [7]. Notably, after the first relapse of a temperate phenotype, multiple subsequent relapses are often observed to occur rapidly [6,9]. In some regions such as India and Central America [10], intermediate phenotypes, or both phenotypes, are present.

Following a bite from a *P. vivax* infectious mosquito, sporozoites are inoculated into the human skin and travel to the liver where they invade hepatocytes. A proportion of these sporozoites will immediately undergo hepatic development giving rise to primary blood-stage infection 9–10 days later [11]. Some sporozoites will transform into hypnozoites, remaining silent and undetectable in the liver for weeks to months until they resume development to cause relapses [7]. The mechanisms regulating hypnozoite activation remain unknown [12], although it has been proposed that relapses may be triggered by fevers from other infections [6,13] or exposure to mosquito bites [14]. Many of the patterns of observed relapse timings are consistent with hypnozoites activating at a constant rate, without the need for external triggers [15].

The hypnozoite activation rate and resulting relapse frequency will influence the transmission potential of *P. vivax*. For tropical strains of *P. vivax*, slow-relapsing strains may be outcompeted by faster-relapsing strains. However, if hypnozoites activate very quickly, then the resulting blood-stage infection may coincide with blood-stage parasites from the primary infection. Assuming human-to-mosquito transmission probability remains constant and independent of blood-stage density [16], the potential for transmission to mosquitoes will be optimized by maximizing the expected duration of blood-stage infection, whether from primary infection or relapses. For temperate strains of *P. vivax*, the optimal time to relapse will additionally be affected by the seasonality of mosquito-borne transmission. In such settings, transmission potential is likely to be optimized by strains that remain dormant over the winter months when the potential for blood-stage parasites to be transmitted to mosquitoes is low.

The virulence trade-off hypothesis asserts that pathogens are under selective pressure to optimize transmission potential [17]. For example, it has been argued that the average density of blood-stage malaria infections has evolved to maximize onwards transmission to mosquitoes, but only to the point where the benefit to transmission is not outweighed by increased host mortality [18]. Similarly, the set-point viral load of HIV is under selective pressure to optimize the number of transmission events [19]. Too high a viral load, and infected individuals are likely to die before they can transmit. Too low a viral load, and the probability of transmission per infectious contact is reduced.

We propose that for *P. vivax*, the hypnozoite activation rate and the duration of dormancy (for temperate strains) are under selective pressure to optimize transmission potential in a given environmental niche. We develop mathematical models of malaria transmission accounting for the patterns of relapses arising from hypnozoites, but with the simplifying assumption that variations in blood-stage density are ignored, and test the hypothesis that optimal relapse frequencies depend on the duration of *P. vivax* blood-stage infection and the seasonality and intensity of transmission.

## 2. Material and methods

### (a) Within-host model of *Plasmodium vivax* relapses

Following primary infection with a tropical strain of *P. vivax*, hypnozoites enter a latent stage in the liver where they either activate to cause relapses, or die within liver hepatocytes. An existing within-host model [15] is used to describe the progress of liver-stage infection and the resulting relapse patterns. The model is determined by the following biological parameters:

— *N*: expected number of hypnozoites per infectious mosquito bite,
— *α*: hypnozoite activation rate,
— *μ*: hypnozoite death rate.

Hypnozoite activation in the absence of blood-stage infection leads to the occurrence of a relapse. When hypnozoites activate in the presence of blood-stage infection, a relapse also occurs but it may or may not be detected owing to the existing blood-stage parasites. Notably, hypnozoites are assumed to act independently of one another, and the potential role of external relapse triggers is ignored.

Following primary infection with a temperate strain, hypnozoites enter a long-latent stage where activation to cause relapses does not immediately occur [7]. We refer to this period of long-latency as the dormant stage. After dormancy, we assume that hypnozoites progress to the latent stage where activation is possible. There is much uncertainty related to the biology of temperate strains of *P. vivax* relapses. Most notably, the biological mechanisms responsible for the period of dormancy before the first relapse are poorly understood [12]. Two hypotheses that have been proposed are that hypnozoites spend predetermined intervals in the dormancy stage before relapses can occur (e.g. an epigenetic clock) [20,21], or that hypnozoites are activated by some external trigger [6,13]. Here we develop the mathematical details of the epigenetic clock model where hypnozoites must initially undergo a dormancy stage before switching to a latency stage where they can relapse.

Characterizing the extended period of time before first relapse of temperate strains of *P. vivax* relapses requires an additional parameter:

— *d*: duration of dormancy.

During dormancy, it is assumed that hypnozoites cannot activate to cause relapses. However, we assume that hypnozoites may still die owing to hepatocyte death [22]. The duration of dormancy of temperate strains can be described by a number of distributions [8,23]. Here we test cases where it follows an exponential distribution with rate parameter *δ* = 1/*d*, or a gamma distribution with mean *d* and standard deviation *σ_d_* (see the electronic supplementary material for details).

### (b) Binary model of *Plasmodium vivax* relapses

A within-host model can track hypnozoites in the liver, accounting for how relapse patterns depend on hypnozoite numbers. However, tracking hypnozoite numbers as opposed to binary infection status substantially increases analytic complexity. We can formulate a simpler relapse model with hypnozoite infection of the liver viewed as a binary process. The epidemiology of tropical strains of *P. vivax* can then be described by three key parameters:

— *f*: relapse frequency (⇔ 1/time to next relapse),
— *h*: number of relapses per primary infection, and
— *γ*_L_: rate of clearance of liver-stage infection.

Using relationships derived by White *et al.* [15], the parameters describing hypnozoite infection as a binary state can be expressed in terms of the within-host parameters of hypnozoite biology.2.1
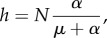
2.2
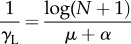
2.3
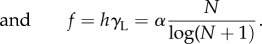


The above equations capture the trade-offs between the parameters for the epidemiology of *P. vivax* relapses. In particular, equation (2.3) assumes a constant relapse rate, e.g. the expected time to first relapse [7] is the same as the expected time between the first and second relapses. This is in contrast to the within-host model [15], which accounts for the increasing time between subsequent relapses owing to the depletion of hypnozoites in the liver [6,24].

Similar to the within-host model, the duration of dormancy of temperate strains is described by the parameter *d*. Relapses are not allowed to occur during the period of dormancy, however liver-stage infection can be cleared as hypnozoites may still die owing to hepatocyte death [22]. This results in a slower rate of clearance of infection during dormancy. Similar to the expression for *γ*_L_ in equation (2.2), the rate of clearance of liver-stage infection during dormancy is2.4
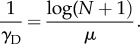


### (c) Mathematical models of malaria transmission

**Figure 1.**
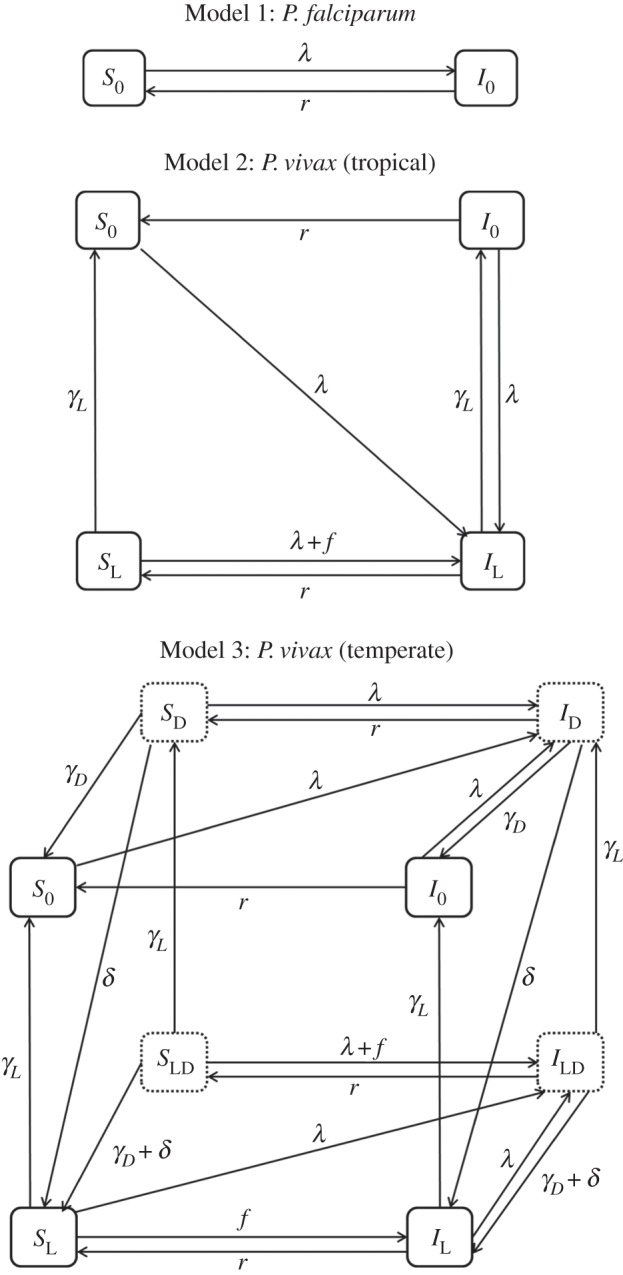
Schematic diagrams of malaria transmission models. The force of infection owing to new mosquito bites is *λ* = *mabI*_M_. Parameter definitions and values are provided in table 1. Model 1: non-relapsing *P. falciparum* malaria. Model 2: relapsing *P. vivax* malaria of a tropical phenotype. Model 3: relapsing *P. vivax* malaria of a temperate phenotype. Dashed boxes denote states where an individual is harbouring dormant hypnozoites. The models are built up by sequentially adding compartments for latent hypnozoite stages, and compartments for dormant hypnozoite stages. Mosquito compartments are not shown.

Analysis of the transmission dynamics of non-relapsing *P. falciparum* malaria is grounded in the theory of Ross-MacDonald models [25,26]. Here, we describe how Ross-MacDonald models can be expanded to account for relapses characteristic of tropical and temperate strains of *P. vivax*. The compartmental model for *P. falciparum* transmission can be extended to incorporate the latent stages for relapses of tropical strains of *P. vivax* (figure 1). This model can be further extended to incorporate the dormant stages characteristic of temperate strains of *P. vivax*.

**Table 1.** Description of model parameters. Parameters are based on estimates from data on temperate strains from Central America [27]. The parameters for the epidemiology of relapses describe the outcome following a single infectious bite.

parameter	description	value	reference
humans			
* b*	transmission probability: mosquito to human	0.5	[28]
* r*	rate of clearance of blood-stage infections	1/60 day^−1^	[29]
* N*	number of hypnozoites per infection	6.4	
* α*	rate of hypnozoite activation	1/230 day^−1^	
* μ*	rate of hypnozoite death	1/217 day^−1^	
* d*	duration of temperate dormancy (*δ* = 1/*d*)	162 days	
* σ_d_*	standard deviation of temperate dormancy	49 days	
* K*	compartments for Gamma distribution 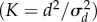	11	
* h*	expected number of relapses	2.1	
* γ*_L_	rate of hypnozoite clearance (latent stage)	1/223 day^−1^	
* f*	relapse frequency (1/time to next relapse)	1/72 day^−1^	
* γ*_D_	rate of hypnozoite clearance (dormant stage)	1/434 day^−1^	
mosquitoes			
* a*	mosquito biting frequency	0.21 day^−1^	[30]
* g*	mosquito death rate (1/mosquito life expectancy)	0.1 day^−1^	[1]
* m*	number of mosquitoes per human	varied	
* n*	duration of sporogony in mosquito	12 days	[1]
* c*	transmission probability: human to mosquito	0.23	[31]

#### (i) Model 1: *Plasmodium falciparum*

Humans are assumed to be in one of two states: susceptible (*S*_0_) or infected (*I*_0_). Mosquitoes are assumed to be in one of two states: susceptible (*S*_M_) or infectious (*I*_M_). Mosquitoes that are infected but not yet sporozoite positive are considered susceptible. The force of infection on humans is given by *λ* = *mabI*_M_. A schematic diagram of the model is presented in figure 1 and equations are presented in the electronic supplementary material. The basic reproduction number for *P. falciparum* malaria [32] as described by this model is2.5
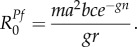


#### (ii) Model 2: *Plasmodium vivax* (tropical)

The mathematical model for *P. falciparum* transmission outlined in figure 1 can be extended to incorporate relapses of tropical strains of *P. vivax* through the addition of states for latent hypnozoites. These states are denoted through sub-script *L*. Following an infectious bite, an individual will develop blood-stage infection (from sporozoites that develop immediately) and latent liver-stage infection (from sporozoites that transform into hypnozoites). Blood-stage infections clear at rate *r*. Liver-stage infections clear at rate *γ*_L_. Liver-stage infection is assumed to cause new blood-stage infections through relapses at rate *f*. It is assumed that relapses do not extend the duration of existing blood-stage infections. The model is described by the following system of differential equations:2.6
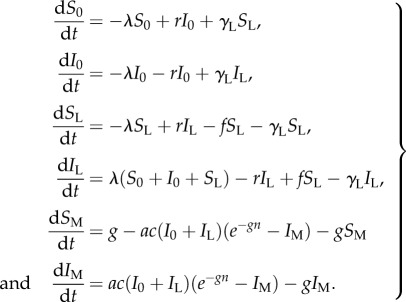
The basic reproduction number will be proportional to the expected time with blood-stage parasites owing to both primary and relapse infections, and is given by2.7
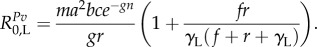


See the electronic supplementary material for details of the derivation. If there are no relapses (i.e. *f* = 0) then equation (2.7) reduces to equation (2.5).

#### (iii) Model 3: *Plasmodium vivax* (temperate)

The equations describing tropical strains of *P. vivax* can be extended to incorporate the dormancy stage characteristic of temperate strains. Following an infectious mosquito bite, an individual will develop blood-stage infection and dormant liver-stage infection (from sporozoites that transform into hypnozoites). These hypnozoites will remain in the dormant stage for an expected duration of *d* days, during which time relapse is not possible. During dormancy, liver-stage infections may be cleared by natural hepatocyte death at rate *γ*_D_. After dormancy, liver-stage infection progresses to the latent stage where hypnozoites can activate to cause relapses at rate *f*. Latent liver-stage infections are assumed to clear at rate *γ*_L_.

If we assume the duration of dormancy is exponentially distributed, then the basic reproduction number is2.8
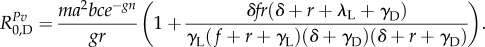


As the duration of dormancy approaches zero, equation (2.8) simplifies to equation (2.7), i.e. as 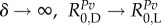. Similar expressions can be derived when the duration of dormancy follows a Gamma distribution [33].

### (d) Seasonality in transmission

Seasonality in exposure to mosquitoes is accounted for using a previously described periodic functional form [34]. The degree of seasonality is measured as the proportion of exposure to mosquitoes occurring in the peak three months. The expressions for *R*_0_ in equations [2.5,2.7,2.8] do not apply in seasonal settings, but can be calculated numerically using Floquet theory [35]—see the electronic supplementary material.

### (e) Strain competition model

A method for modelling competition between many-strain pathogens [36] was adapted for the malaria transmission models in figure 1. The dynamics of co-circulating strains of *P. vivax* with varying time to first relapse were simulated. Competition between strains was mediated via cross-strain immunity whereby the probability of infection with new strains reduces with the multiplicity of infection [37].

### (f) Model parametrization

The within-host model was fitted to data from six cohorts where participants were followed longitudinally for the detection of relapses following primary *P. vivax* infection from either artificial challenge or natural exposure [24,27,38–41]. Up to five relapses were observed per individual, with treatment administered following each infection. We assume that new blood-stage infections are not possible for 14 days after each new relapse, based on the duration of prophylactic protection of the administered treatment.

The models were fitted to the data using Approximate Bayesian Computation (ABC). This method was preferred over likelihood-based methods owing to the difficulty in constructing likelihoods that account for unobserved events during the observation period. The acceptance criteria for the ABC algorithm was based on agreement between model predictions and 95% CIs from survival analysis of the data. Further details are in the electronic supplementary material.

## 3. Results

### (a) Epidemiological parameters of *Plasmodium vivax* relapses

**Figure 2.**
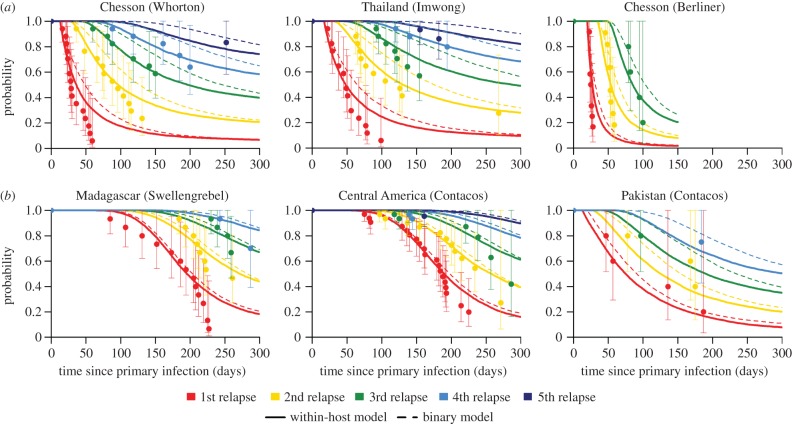
(*a*) Tropical and (*b*) temperate relapse phenotypes. Comparison between model predicted survival time until *n*th relapse and data from six cohorts. After primary infection, participants in each cohort were followed longitudinally for the detection of relapses. Up to five relapses were detected per participant with treatment administered following each detected relapse. Data are presented using survival analysis to show the proportion of individuals with at least *n* relapses (circles) and 95% confidence intervals (vertical lines). The duration of dormancy for the temperate relapse phenotype is described by a gamma distribution. The solid curves shows the best-fit prediction from the within-host model (number of hypnozoites accounted for) and the dashed curves denote the prediction from the binary model (hypnozoite infection regarded as a binary state). The close agreement between the solid and dashed curves suggests that the within-host biology of hypnozoites and subsequent relapses can be approximated by simpler binary models. All parameter values are provided in the electronic supplementary material.

Figure 2 shows examples of model fits to datasets from tropical relapse phenotypes (Model 2, figure 2*a*) and temperate relapse phenotypes (Model 3, figure 2*b*). The best-fit parameters for the within-host model are provided in the electronic supplementary material. The estimated within-host parameters were used to obtain estimates of the relapse parameters for the binary infection model described in equations (1–4). The relapse timings predicted by the binary model are also plotted in figure 2. The close agreement between predicted relapse timings in figure 2 demonstrates that the relapse patterns predicted by detailed within-host models [15] can be approximated by the simpler binary models from figure 1. Notably, the time to next relapse is memoryless for the binary model and does not depend on the number of previous relapses. However, for the within-host model, the time to next relapse depends on the number of hypnozoites in the liver and hence the number of past relapses.

### (b) Optimizing the transmission potential of *Plasmodium vivax* in non-seasonal settings

**Figure 3.**
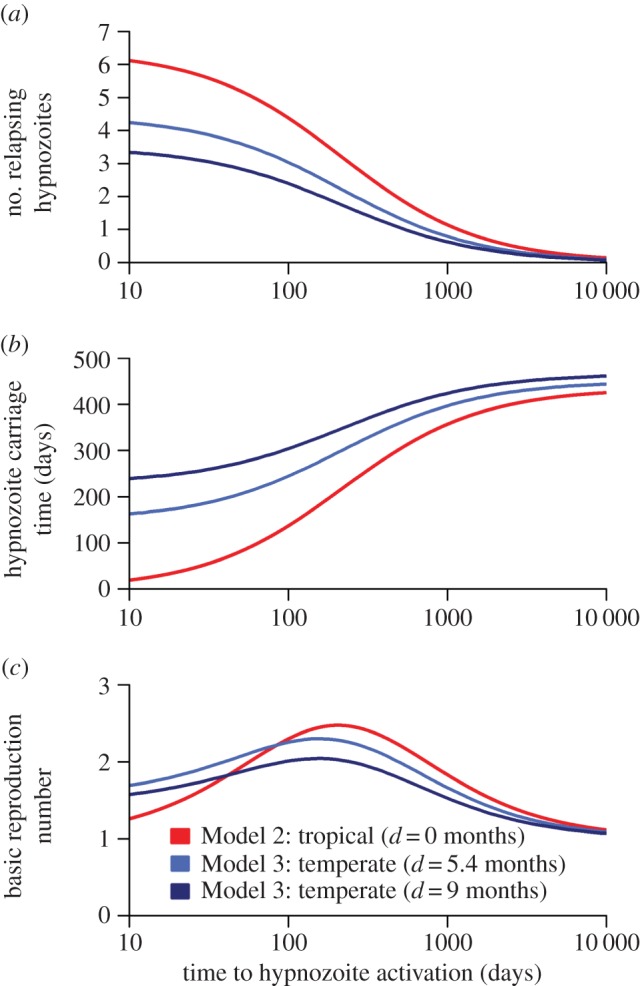
The optimal transmission potential of *P. vivax* in non-seasonal settings. (*a*) Slower hypnozoite activation rates reduce the number of relapsing hypnozoites. This is because spending more time in the liver increases the probability of hypnozoite death. (*b*) Slower hypnozoite activation rates increase the expected duration of time spent by hypnozoites in the liver. In particular, very fast relapse rates lead to hypnozoites being flushed out of the liver and short durations of hypnozoite carriage. (*c*) Transmission potential (as measured by *R*_0_) is optimized at intermediate values of relapse frequency, here predicted to be *α* = 1/206 days for tropical strains. Relapse too quickly and all relapses coincide with the primary infection; relapse too slowly and hypnozoites risk death in the liver.

Figure 3*a* shows how the expected number of relapsing hypnozoites is predicted to decrease with slower hypnozoite activation rates. Note that if several hypnozoites activate within a short period of time (e.g. a few days) they may be classified as a single relapse. Figure 3*b* shows how the duration of time spent in the liver increases for slower hypnozoite activation rates. Figure 3*c* shows how *R*_0_ is optimized at intermediate values of *α* (corresponding to intermediate relapse frequencies), where there is a balance between hypnozoites waiting for the primary infection to clear before relapsing, but not waiting too long to risk death in liver hepatocytes [22]. This maximizes the time that transmissible parasites spend in the blood (arising from either primary infection or relapses). For tropical strains, *R*_0_ is maximized when the hypnozoite activation rate *α* ≈ 1/206 days, corresponding to a relapse frequency of *f* ≈ 1/64 days. A similar pattern is observed for temperate strains, although increasing the duration of dormancy reduces the expected number of relapsing hypnozoites, and increases the duration of liver-stage infection.

### (c) Variation of transmission potential of *Plasmodium vivax* in seasonal settings

**Figure 4.**
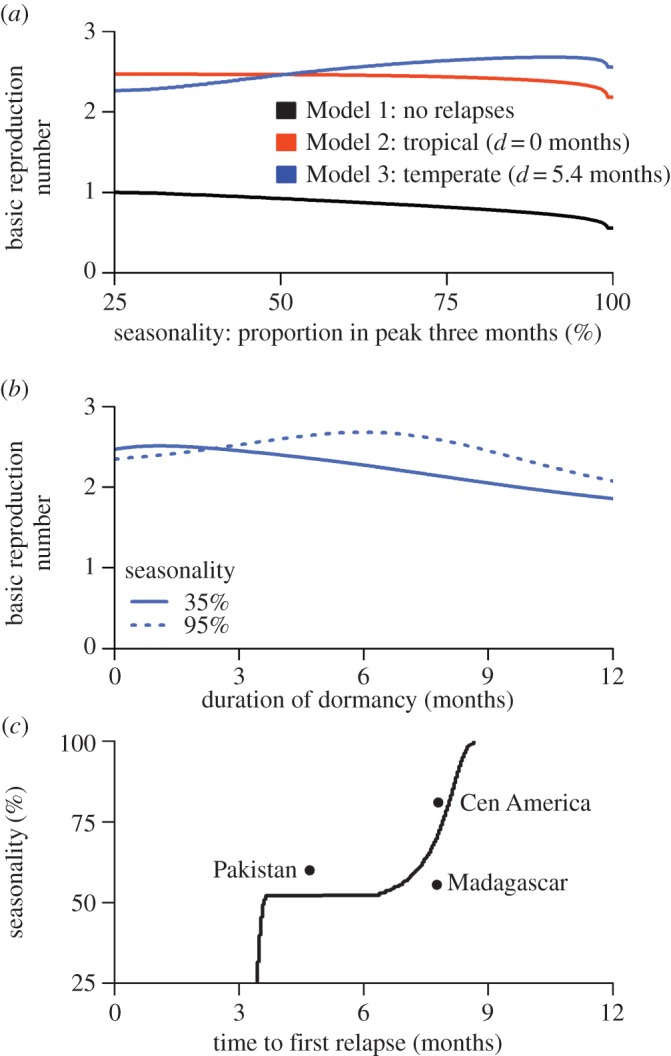
Effect of seasonality on the transmission potential of *P. vivax*. (*a*) For non-relapsing *P. falciparum*, *R*_0_ decreases with increasing seasonality. For tropical strains of *P. vivax*, there is a modest reduction in *R*_0_ with increasing seasonality. By contrast, for temperate strains of *P. vivax*, *R*_0_ increases with seasonality. In the simulations presented here, the difference between *R*_0_ for *P. falciparum* and *P. vivax* is owing to relapses. (*b*) The transmission potential of temperate strains of *P. vivax* will depend on the duration of dormancy, with *R*_0_ optimized at longer durations in more seasonal settings. (*c*) For a given seasonal profile, the time to first relapse (dormancy plus latency in the liver) can be estimated by maximizing *R*_0_. The time to first relapse (solid line) is predicted to increase with seasonality. Notably when seasonality crosses a threshold of ≈50% of transmission in the peak three months, the time to first relapse switches from 3–4 months characteristic of tropical phenotypes [24,38,39] to 6–9 months characteristic of temperate phenotypes [27,40].

Increasing the degree of seasonality of malaria transmission is predicted to reduce *R*_0_ for non-relapsing *P. falciparum* (figure 4*a*). Tropical strains of *P. vivax* are predicted to exhibit modest variation in transmission potential with variation in seasonality. By contrast, the transmission potential of temperate strains of *P. vivax* is predicted to increase in more seasonal settings. Figure 4*b* shows how the transmission potential of temperate strains of *P. vivax* depends on the duration of dormancy. In more seasonal settings, *R*_0_ is maximized at longer durations of dormancy, such that the time to first relapse coincides with the period between transmission peaks. The model proposed here can be used to predict relapse frequencies for a given seasonal profile (figure 4*c*). For temperate strains of *P. vivax* the duration of dormancy, and hence the time to first relapse, are predicted to increase with seasonality. There is a notable switch in optimal relapse times, from 3–4 months in low-seasonality settings (characteristic of tropical zones), to 7–8 months in high-seasonality settings (characteristic of temperate zones), in agreement with epidemiological observations [7,8,27,40,41].

### (d) Variation in relapse frequency with transmission intensity

**Figure 5.**
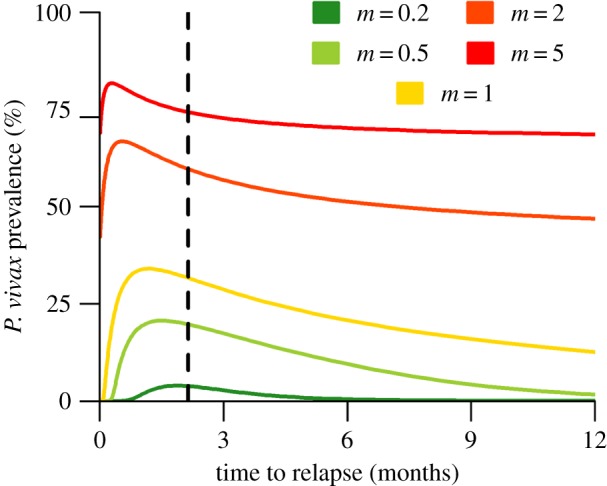
The effect of time to next relapse on equilibrium parasite prevalence of tropical *P. vivax*. Transmission intensity was varied by changing the number of mosquitoes per human *m*. At higher transmission intensities, the time to next relapse that maximizes *P. vivax* prevalence (*Pv*PR) decreases. Faster relapses are favoured at high prevalence because if hypnozoites wait for the primary infection to clear, another primary infection will occur. Slower relapses are favoured at lower prevalence because hypnozoites can safely wait for the primary infection to clear before relapsing. The vertical dashed line corresponds to the time to first relapse (*f* = 66 days) that optimizes *R*_0_.

Optimizing *R*_0_ maximizes the number of secondary infections arising from a single individual in an otherwise susceptible population. However, this may be at the expense of longer generation times (the expected time between primary infection in a human and primary infection in another human after one generation of transmission). For tropical strains of *P. vivax*, figure 5 shows how the predicted equilibrium parasite prevalence (*Pv*PR) varies with relapse frequencies across a range of transmission intensities. At very low transmission intensity when most of the population is susceptible, *Pv*PR is optimized at the same relapse frequency that optimizes *R*_0_. As transmission intensity increases, the time to next relapse that maximizes *Pv*PR decreases. The advantage of waiting in the liver diminishes because a hypnozoite-infected individual may receive another primary infection from a mosquito before the hypnozoite has a chance to relapse. In particular, as transmission intensity increases the time to relapse that maximizes *Pv*PR approaches zero. Conversely, if *P. vivax* transmission is reduced through malaria control interventions, slower-relapsing strains may be selected for.

### (e) Competition between strains of varying relapse frequency

**Figure 6.**
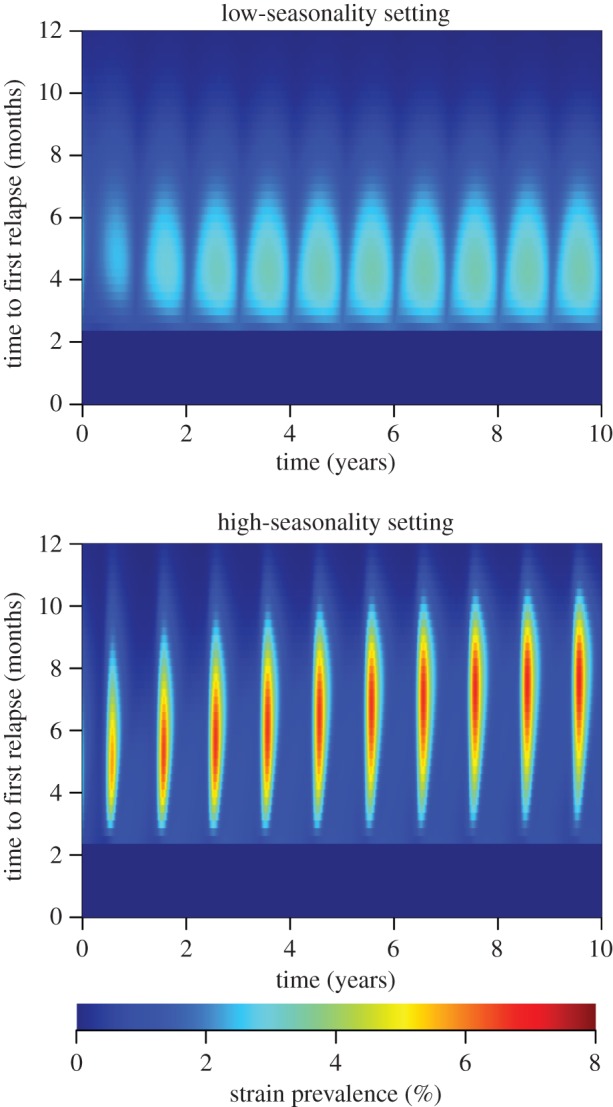
Competition between temperate strains of *P. vivax* with varying time to first relapse. The time to first relapse was varied by changing the duration of dormancy *d* while keeping the average time in the latent stage fixed (1/*f*). Simulations were initialized with the distribution of strain phenotype assumed to follow a normal distribution. In a low seasonality setting, the mean time to first relapse converges on approximately four months. In a high-seasonality setting, the mean time to first relapse converges on six to nine months.

The previous analyses investigated transmission potential in terms of *R*_0_ (figures 3 and 4) or equilibrium parasite prevalence (figure 5), but did not consider direct competition between strains with different relapse frequencies. Figure 6 shows the simulated dynamics of 200 strains of temperate *P. vivax* with varying durations of dormancy *d*. The distribution of *d* in these strains initially followed a zero-truncated normal distribution with a mean of two months and standard deviation of three months. In the low-seasonality setting, fast-relapsing strains are selected, with time to first relapse approaching four months. In the high-seasonality setting, slower-relapsing strains are selected for with the time to first relapse coinciding with the time between consecutive seasonal peaks at around eight months.

## 4. Discussion

Relapses are a key driver of *P. vivax* transmission [15], with the fitness of *P. vivax* strongly influenced by relapse frequency. Here we predict that transmission potential is optimized at intermediate relapse frequencies that balance the benefits of slow-relapsing strains (longer total duration of blood-stage parasitemia) with the benefits of fast-relapsing strains (increased probability that parasites are transmitted quickly before hypnozoites die in liver hepatocytes). This trade-off provides insight into the globally observed variation in *P. vivax* relapse frequency, and the potential effects on relapse frequency of reducing transmission through malaria control.

Seasonality has been recognized as an important driver of the transmission potential of many pathogens [42,43]. Here, we demonstrate how seasonality in exposure to mosquitoes, driven by seasonal fluctuations in temperature and rainfall [1], affects the transmission potential of *P. vivax*. Our analysis predicts that the time to first relapse increases with the degree of seasonality of transmission. Notably, as seasonality increases, there is a sharp switch from short duration of dormancy (tropical phenotype) to a long duration of dormancy (temperate phenotype). This suggests that in areas where *P. vivax* is endemic, circulating strains will be either tropical or temperate, but not both. However, the model did not consider the complex seasonal patterns of transmission observed in places such as India [44], where both phenotypes are present.

This analysis suggest that relapse frequencies depend on the intensity and seasonality of malaria transmission in the absence of relapse triggers such as fevers induced by co-circulating pathogens such as *P. falciparum* [6]—an important limitation. While triggers may add an additional layer of complexity to relapse patterns, their role is unlikely to affect the findings of this analysis. A further limitation relates to the density of blood-stage infections [29]. Although relapses may play a role in increasing blood-stage parasite densities, we assume constant human-to-mosquito transmission probability throughout blood-stage infection, despite evidence that higher gametocyte densities are associated with increased transmission probability [45]. This has implications for the virulence trade-off hypothesis [17], which has been investigated for malaria [18,46]: increasing parasite densities lead to higher probability of transmission to mosquitoes, but also increased host mortality and shorter durations of infection. The trade-off in relapse rates to optimize *P. vivax* transmission potential can be considered a corollary of the virulence trade-off hypothesis. Although not accounted for in the analytic models presented here, the additional complexity arising from within-host processes regulating blood-stage parasites can be addressed using simulation models [16].

In the framework considered here, the transmission potential of *P. vivax* is optimized by maximizing the time that an infected individual spends with blood-stage parasites. The early generation of *P. vivax* gametocytes ensures that onward transmission to mosquitoes is likely throughout blood-stage infection [45]. In an otherwise susceptible population, the conditions for optimal transmission potential are the same as the conditions for maximum *R*_0_. However, in settings with high *P. vivax* prevalence, transmission potential will be affected by primary infections from new mosquito bites. For example, in a high transmission setting an individual infected with a slow-relapsing strain may receive a second infection from a new mosquito bite before the relapse from the primary infection occurs. This causes faster-relapsing strains to be selected for in high transmission settings.

The relationship between time to relapse and seasonality predicted here is in agreement with epidemiological data on the geographical distribution of relapses [7,8]. Furthermore, our model suggests that observed strains have close to optimal fitness for their geographical location, including in regions such as South America where *P. vivax* was reportedly introduced approximately 500 years ago [47]. As transmission is reduced globally through increased malaria control all strains will be targeted, but the timeline to effectiveness will be more rapid for faster-relapsing strains. Indeed, as malaria transmission is progressively reduced, the proportion of slow-relapsing strains may increase. This change may arise as a consequence of the higher transmission potential of existing circulating strains with slow relapse frequency, or from the importation of strains from other regions. Although the de novo evolution of strains with slower relapse frequency is possible, it is unlikely to be significant over the epidemiological timescales of interest. Rapid scale-up of malaria control interventions may target all strains of *P. vivax*, reducing the opportunity for slow-relapsing strains to become established. A comparable argument has been made for the widespread deployment of anti-retroviral treatment for HIV to rapidly reduce HIV transmission before the transmission trade-off can plausibly select strains of increased virulence [19]. Similarly, a strategy of rapid scale-up of malaria control interventions may reduce the risk of an increased proportion of slow-relapsing strains of *P. vivax*. Although fast-relapsing strains may cause greater morbidity in infected individuals, slow-relapsing strains may increase the timeline to malaria elimination in a population with low levels of *P. vivax* transmission long after *P. falciparum* has been sustainably eliminated [48].

## Supplementary Material

Supporting Information

## Data accessibility

Data on relapse timings: Dryad: http://dx.doi.org/10.5061/dryad.mg73v.

## References

[1] Gething PW, Van Boeckel TP, Smith DL, Guerra CA, Patil AP, Snow RW, Hay SI. 2011 Modelling the global constraints of temperature on transmission of *Plasmodium falciparum* and *P. vivax*. Parasites Vectors 4, 92 (10.1186/1756-3305-4-92) 21615906 PMC3115897

[2] Gething PW et al. 2012 A long neglected world malaria map: *Plasmodium vivax* endemicity in 2010. PLoS Neg. Trop. Dis. 6, e1814 (10.1371/journal.pntd.0001814) PMC3435256 22970336

[3] Hulden L, Hulden L 2009 The decline of malaria in Finland—the impact of the vector and social variables. Malar. J. 8, 94 (10.1186/1475-2875-8-94) 19422702 PMC2684538

[4] Betuela I et al. 2012 Epidemiology of malaria in the Papua New Guinean highlands. Trop. Med. Int. Health 17, 1181–1191. (10.1111/j.1365-3156.2012.03062.x) 22925472

[5] Howes RE et al. 2011 The global distribution of the Duffy blood group. Nat. Comm. 2, 266 (10.1038/ncomms1265) PMC3074097 21468018

[6] White NJ 2011 Determinants of relapse periodicity in *Plasmodium vivax* malaria. Malar J. 10, 297 (10.1186/1475-2875-10-297) 21989376 PMC3228849

[7] Battle KE et al. 2014 Geographical variation in *Plasmodium vivax* relapse. Malar J. 13, 144 (10.1186/1475-2875-13-144) 24731298 PMC4021508

[8] Lover AA, Coker RJ 2013 Quantifying effect of geographic location on epidemiology of *Plasmodium vivax* malaria. Emerg. Inf. Dis. 19, 1058–1065. (10.3201/eid1907.121674) PMC3713979 23763820

[9] Hankey DD, Jones R Jr, Coatney GR, Alving AS, Coker WG, Garrison PL, Donovan WN 1953 Korean vivax malaria. I. Natural history and response to chloroquine. Am. J. Trop. Med. Hyg. 2, 958–969. 13104804

[10] Roy M, Bouma MJ, Ionides EL, Dhiman RC, Pascual M 2013 The potential elimination of *Plasmodium vivax* malaria by relapse treatment: insights from a transmission model and surveillance data from NW India. PLoS Neg. Trop. Dis. 7, e1979 (10.1371/journal.pntd.0001979) PMC3542148 23326611

[11] Mikolajczak SA et al. 2015 *Plasmodium vivax* liver stage development and hypnozoite persistence in human liver-chimeric mice. Cell Host Microbe 17, 526–535. (10.1016/j.chom.2015.02.011) 25800544 PMC5299596

[12] Mueller I, Galinski MR, Baird JK, Carlton JM, Kochar DK, Alonso PL, del Portillo HA. 2009 Key gaps in the knowledge of *Plasmodium vivax*, a neglected human malaria parasite. Lancet Inf. Dis. 9, 555–566. (10.1016/S1473-3099(09)70177-X) 19695492

[13] Shanks GD, White NJ 2013 The activation of *vivax* malaria hypnozoites by infectious diseases. Lancet Inf. Dis. 13, 900–906. (10.1016/S1473-3099(13)70095-1) 23809889

[14] Hulden L, Hulden L 2011 Activation of the hypnozoite: a part of *Plasmodium vivax* life cycle and survival. Malar J. 10, 90 (10.1186/1475-2875-10-90) 21496287 PMC3086824

[15] White MT, Karl S, Battle KE, Hay SI, Mueller I, Ghani AC 2014 Modelling the contribution of the hypnozoite reservoir to *Plasmodium vivax* transmission. eLife 3, 411 (10.7554/eLife.04692) PMC4270097 25406065

[16] Greischar MA, Reece SA, Mideo N 2015 The role of models in translating within-host dynamics to parasite evolution. Parasitology. (10.1017/S0031182015000815) PMC4877521 26399436

[17] Alizon S, Hurford A, Mideo N, Van Baalen M 2009 Virulence evolution and the trade-off hypothesis: history, current state of affairs and the future. J. Evol. Biol. 22, 245–259. (10.1111/j.1420-9101.2008.01658.x) 19196383

[18] Mackinnon MJ, Read AF 2004 Virulence in malaria: an evolutionary viewpoint. Phil. Trans. R. Soc. Lond. B 359, 965–986. (10.1098/rstb.2003.1414) 15306410 PMC1693375

[19] Fraser C, Lythgoe K, Leventhal GE, Shirreff G, Hollingsworth TD, Alizon S, Bonhoeffer S. 2014 Virulence and pathogenesis of HIV-1 infection: an evolutionary perspective. Science 343, 1328 (10.1126/science.1243727) PMC5034889 24653038

[20] Barnwell JW, Galinski MR 2014 Malarial liver parasites awaken in culture. Nat. Med. 20, 237–239. (10.1038/nm.3498) 24603792

[21] Chen N, Auliff A, Rieckmann K, Gatton M, Cheng Q 2007 Relapses of *Plasmodium vivax* infection result from clonal hypnozoites activated at predetermined intervals. J. Inf. Dis. 195, 934–941. (10.1086/512242) 17330782

[22] Malato Y, Naqvi S, Schuermann N, Ng R, Wang B, Zape J, Kay MA, Grimm D, Willenbring H. 2011 Fate tracing of mature hepatocytes in mouse liver homeostasis and regeneration. J. Clin. Invest. 121, 4850–4860. (10.1172/JCI59261) 22105172 PMC3226005

[23] Lover AA, Zhao X, Gao Z, Coker RJ, Cook AR 2014 The distribution of incubation and relapse times in experimental human infections with the malaria parasite *Plasmodium vivax*. BMC Inf. Dis. 14, 539 (10.1186/1471-2334-14-539) PMC4287165 25280926

[24] Berliner RW, Earle DP, Taggart JV, Welch WJ, Zubrod CG, Knowlton P, Atchley JA, Shannon JA. 1948 Studies on the chemotherapy of the human malarias. VII. The antimalarial activity of pamaquine. J. Clin. Invest. 27, 108–113. (10.1172/JCI101947) PMC438898 16695621

[25] Macdonald G 1952 The analysis of equilibrium in malaria. Trop. Dis. Bull. 49, 813–829. 12995455

[26] Smith DL, Battle KE, Hay SI, Barker CM, Scott TW, McKenzie FE 2012 Ross, Macdonald, and a theory for the dynamics and control of mosquito-transmitted pathogens. PLoS Path. 8, e1002588 (10.1371/journal.ppat.1002588) PMC3320609 22496640

[27] Contacos PG, Collins WE, Jeffery GM, Krotoski WA, Howard WA 1972 Studies on characterization of *Plasmodium vivax* strains from Central America. Am. J. Trop. Med. Hyg. 21, 707. 4627547 10.4269/ajtmh.1972.21.707

[28] Smith DL, Drakeley CJ, Chiyaka C, Hay SI 2010 A quantitative analysis of transmission efficiency versus intensity for malaria. Nat. Comm. 1, 108 (10.1038/ncomms1107) PMC3065713 21045826

[29] Kerlin DH, Gatton ML 2015 A simulation model of the within-host dynamics of *Plasmodium vivax* infection. Malar J. 14, 51 (10.1186/s12936-015-0580-z) 25652017 PMC4323116

[30] Garrett-Jones C 1964 The human blood index of malaria vectors in relation to epidemiological assessment. Bull. World Health Organ. 30, 241–261. 14153413 PMC2554803

[31] Bharti AR, Chuquiyauri R, Brouwer KC, Stancil J, Lin J, Llanos-Cuentas A, Vinetz JM 2006 Experimental infection of the neotropical malaria vector *Anopheles darlingi* by human patient-derived *Plasmodium vivax* in the Peruvian Amazon. Am. J. Trop. Med. Hyg. 75, 610–606. 17038681 PMC1630632

[32] Smith DL, McKenzie FE, Snow RW, Hay SI 2007 Revisiting the basic reproductive number for malaria and its implications for malaria control. PLoS Biol. 5, 531–542 (10.1371/journal.pbio.0050042) PMC1802755 17311470

[33] Wearing HJ, Rohani P, Keeling MJ 2005 Appropriate models for the management of infectious diseases. PLoS Med. 2, 621–627. (10.1371/journal.pmed.0020174) PMC1181873 16013892

[34] Griffin JT 2015 The interaction between seasonality and pulsed interventions against malaria in their effects on the reproduction number. PLoS Comp. Biol. 11, e1004057 (10.1371/journal.pcbi.1004057) PMC4295870 25590612

[35] Bacaer N 2007 Approximation of the basic reproduction number *R*_0_ for vector-borne diseases with a periodic vector population. Bull. Math. Biol. 69, 1067–1091. 17265121 10.1007/s11538-006-9166-9

[36] Gog JR, Grenfell BT 2002 Dynamics and selection of many-strain pathogens. Proc. Natl Acad. Sci. USA 99, 17 209–17 214. (10.1073/pnas.252512799) PMC139294 12481034

[37] Recker M, Nee S, Bull PC, Kinyanjui S, Marsh K, Newbold C, Gupta S. 2004 Transient cross-reactive immune responses can orchestrate antigenic variation in malaria. Nature 429, 555–558. (10.1038/nature02486) 15175751

[38] Whorton CM, Yount E Jr 1947 The Chesson strain of *Plasmodium vivax* malaria; clinical aspects. J. Inf. Dis. 80, 237–249. (10.1093/infdis/80.3.237) 20247931

[39] Imwong M, Boel ME, Pagornrat W, Pimanpanarak M, McGready R, Day NPJ, Nosten F, White NJ. 2012 The first *Plasmodium vivax* relapses of life are usually genetically homologous. J. Inf. Dis. 205, 680–683. (10.1093/infdis/jir806) 22194628 PMC3266132

[40] Swellengrebel NH, de Buck A 1932 Plasmoquine prophylaxis in benign tertian malaria. Proc. R. Acad. Amsterdam 35, 911–914.

[41] Contacos PG, Coatney GR, Collins WE, Briesch PE, Jeter MH 1973 5 day primaquine therapy—evaluation of radical curative activity against *vivax* malaria infection. Am. J. Trop. Med. Hyg. 22, 693–695. 4583162 10.4269/ajtmh.1973.22.693

[42] Cornet S, Nicot A, Rivero A, Gandon S 2014 Evolution of plastic transmission strategies in avian malaria. PLoS Path. 10, e1004308 (10.1371/journal.ppat.1004308) PMC4161439 25210974

[43] Koelle K, Pascual M, Yunus M 2005 Pathogen adaptation to seasonal forcing and climate change. Proc. R. Soc. B 272, 971–977. (10.1098/rspb.2004.3043) PMC1564099 16024354

[44] Adak T, Sharma VP, Orlov VS 1998 Studies on the *Plasmodium vivax* relapse pattern in Delhi, India. Am. J. Trop. Med. Hyg. 59, 175–179. 9684649 10.4269/ajtmh.1998.59.175

[45] Bousema T, Drakeley C 2011 Epidemiology and infectivity of *Plasmodium falciparum* and *Plasmodium vivax* gametocytes in relation to malaria control and elimination. Clin. Microbiol. Rev. 24, 377–410. (10.1128/CMR.00051-10) 21482730 PMC3122489

[46] Mackinnon MJ, Gandon S, Read AF 2008 Virulence evolution in response to vaccination: the case of malaria. Vaccine 26, C42–C52. (10.1016/j.vaccine.2008.04.012) 18773536 PMC2663389

[47] Carter R 2003 Speculations on the origins of *Plasmodium vivax* malaria. Trends Parasitol. 19, 214–219. (10.1016/S1471-4922(03)00070-9) 12763427

[48] Smith DL et al. 2013 A sticky situation: the unexpected stability of malaria elimination. Phil. Trans. R. Soc. B 368, 20120145 (10.1098/rstb.2012.0145) 23798693 PMC3720043

