# Supplementary material for: Variation in relapse frequency and the transmission potential of Plasmodium vivax malaria
Source: Proc Biol Sci. 2016 Mar 30;283(1827):20160048. doi: 10.1098/rspb.2016.0048 (PMC4822465; doi:10.1098/rspb.2016.0048)
Supplement: Supporting Information [file rspb20160048supp1.docx]

***Supporting Information for***

**Variation in relapse frequency and the transmission potential of *Plasmodium vivax* malaria**

Michael White*, George Shirreff, Stephan Karl, Azra Ghani, Ivo Mueller

*Corresponding author: [m.white08@imperial.ac.uk](mailto:m.white08@imperial.ac.uk)

**Summary**

In this supplement, we provide details of how the parameters for the within-host model were estimated from data using Approximate Bayesian Computation (ABC) methods. We also provide full details of the differential equations used for the malaria transmission models. For each transmission model, a derivation of the basic reproduction number is provided, in both seasonal and non-seasonal settings. We also provide additional details of how the basic reproduction number is optimised with respect to the relapse parameters.

**1. A framework for classifying *P. vivax* relapses**

In the models presented in Figure 1, *P. vivax* is classified into two phenotypes according to patterns of relapses. Tropical phenotypes (Model 2) are assumed to produce hypnozoites that can activate at any time during liver-stage latency to initiate relapses. Temperate phenotypes (Model 3) are assumed to produce hypnozoites that first undergo a period of dormancy in the liver before progressing to a latent liver-stage where activation to cause relapses is possible. Although this classification of relapse phenotypes has been widely used, it has long been recognised that it is not sufficient in all cases (6). For example, in temperate Korea, *P. vivax* infections have been observed with no primary infection and the first relapse not occurring until at least 6 months (9). Epidemiological observations of
*P. vivax* infections from India suggest that relapses follow both the tropical and temperate patterns (40). Whether this is due to co-circulating tropical and temperate strains, or a single strain with a plastic phenotype is unclear.

Figure S1 provides a suggested framework for classifying *P. vivax* relapse phenotypes according to the within-host behaviour of sporozoites and hypnozoites. Two key parameters are required to classify the type of infection:

- *p*: proportion of sporozoites developing into hypnozoites
- *q*: proportion of hypnozoites entering the dormant stage where activation cannot occur immediately

In order for relapses to occur, some hypnozoites must develop (*p* > 0). For tropical strains of *P. vivax*, hypnozoites enter the latent stage where activation can occur immediately (*q* = 0). For temperate strains, some or all hypnozoites enter a dormant stage where they must wait before progressing to the latent stage where activation can occur (*q* > 0). Thus temperate strains have a dormant stage but tropical strains do not. Table S1 describes how choice of parameter values can be used to classify relapse phenotype.

**
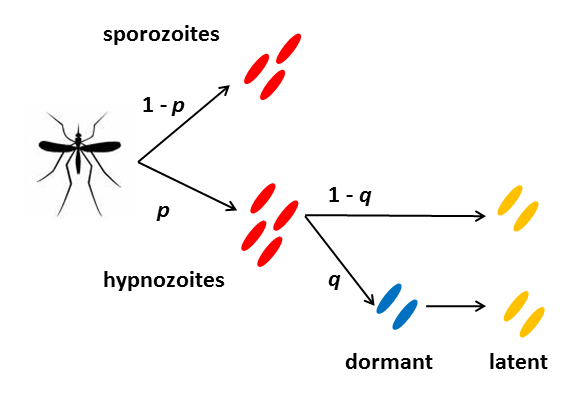
**

Figure S1: A framework for classifying *P. vivax* infections. Sporozoites are inoculated following a bite from an infectious mosquito. A proportion 1 - *p* undergo immediate development leading to a primary infection. A proportion *p* develop into hypnozoites, which can be in a latent stage where activation to cause relapses is possible (characteristic of tropical strains), or a dormant stage where they cannot activate until they progress to the latent stage after some duration of time (characteristic of temperate strains).

Table S1. Parameter ranges for the framework in Figure S1 corresponding to *P. falciparum* and several *P. vivax* phenotypes.

| **strain** | **hypnozoite**  **proportion** | **dormant proportion** |
| --- | --- | --- |
| Model 1: *P. falciparum* | *p*  = 0 |  |
| Model 2: *P. vivax* (tropical) | 0 < *p* < 1 | *q* = 0 |
| Model 3: *P. vivax* (temperate) | 0 < *p* < 1 | *q* = 1 |
| Model 3: *P. vivax* (temperate – Korean) | *p* = 1 | *q* = 1 |
| Model 3: *P. vivax* (mixed – Indian) | 0 < *p* < 1 | 0 < *q* < 1 |

**2. Statistical methods for estimating relapse parameters**

**2.1. Data on multiple relapses**

The within-host relapse model (15) was fitted to data on relapses of both tropical and temperate strains of *P. vivax*. Six studies where participants were followed up for multiple relapses after primary infection were identified and are summarised in Table S2. Following detection of a primary or relapse infection participants were treated. We assume a censoring period of *T_cen_* = 14 days following primary and relapse infections. Relapses may occur during this censoring period but will go undetected. Participants were challenged with the bites from multiple infectious mosquitoes. We assume that each individual is exposed to *ν* infectious bites, each giving rise to an average of *N* hypnozoites. We assume that the number of bites from a single mosquito follows a geometric distribution with mean *N*. The number of hypnozoites from *ν* will therefore follow a negative binomial distribution with mean *νN* and failure parameter *ν*.

Table S2: Overview of datasets included for fitting the within-host model.

| **Study** | **Location** | **Strain** | **Phenotype** | **Participants** | **Max relapses** | **Total relapses** |
| --- | --- | --- | --- | --- | --- | --- |
| Whorton (33) | USA | Chesson | tropical | 17 | 5 | 43 |
| Imwong (34) | Thailand | Thailand (wild) | tropical | 17 | 5 | 39 |
| Berliner (24) | USA | Chesson | tropical | 12 | 3 | 28 |
| Swellengrebel (35) | Holland | Madagascar | temperate | 15 | 4 | 30 |
| Contacos (37) | USA | Central America | temperate | 38 | 5 | 43 |
| Contacos (36) | USA | Pakistan | temperate | 5 | 4 | 12 |

**2.2. Approximate Bayesian Computation (ABC) algorithm**

For each dataset, the proportion of participants who have experienced at least *j* relapses at a given time was calculated using a Kaplan-Meier estimate of the survival function accounting for right-censoring. The point estimates and 95% confidence intervals of the data were calculated using the survfit function from the survival library in R (49). The data and 95% confidence intervals are presented in Figure S2. The data is described with the following notation:

$$X_{j}^{t}=\text{Prob }\leq j\text{ relapses at time }t$$

With 95% confidence intervals calculated via survival analysis defined to be$X_{j,2.5\%}^{t}$ and$X_{j,97.5\%}^{t}$.

The within-host relapse model was used to stochastically simulate relapse times for either the tropical or temperate phenotype. For the tropical phenotype the parameter vector $\theta=\left(N,\nu,\alpha,\mu\right)$ is used to simulate relapse times. For the temperate phenotype the parameter vector $\theta=\left(N,\nu,\alpha,\mu,d,\sigma_{d}\right)$ is used to simulate relapse times. For a cohort with *P* paticipants, we obtain a stochastic simulation for the proportion of individuals with at least *j* relapses by time *t*, denoted $Y_{j}^{t}$. The routine for stochastic simulation of $Y_{j}^{t}$ is described below in Section 2.4.

The within-host model for relapse timings was fitted to the data using Approximate Bayesian Computation (ABC) methods. This method was preferred over likelihood based methods due to the difficulty in constructing likelihoods that account for unobserved events during the censoring period.

For each model and dataset, 1,000,000 parameters were randomly sampled from the prior distributions described in Table S3. For each parameter set the model prediction of $Y_{j}^{t}$ was stochastically simulated. The sampled parameter was accepted if and only if

$$X_{j,2.5\%}^{t}\leq Y_{j}^{t}\leq X_{j,97.5\%}^{t}\text{ }\forall j,\forall t$$

The distribution of the accepted parameters provides an approximation of the posterior distribution. Posterior medians and 95% credible intervals are provided in Table S3.

**2.3. Model fits to data**

Figure S2 shows a comparison between model predicted times to relapse and the datasets described in Table S2. The parameters for model simulations are taken to be the medians of the posterior distributions (Table S3). Two models were fitted to data on temperate strains of *P. vivax*: one with exponentially distributed dormancy, and one with gamma distributed dormancy.


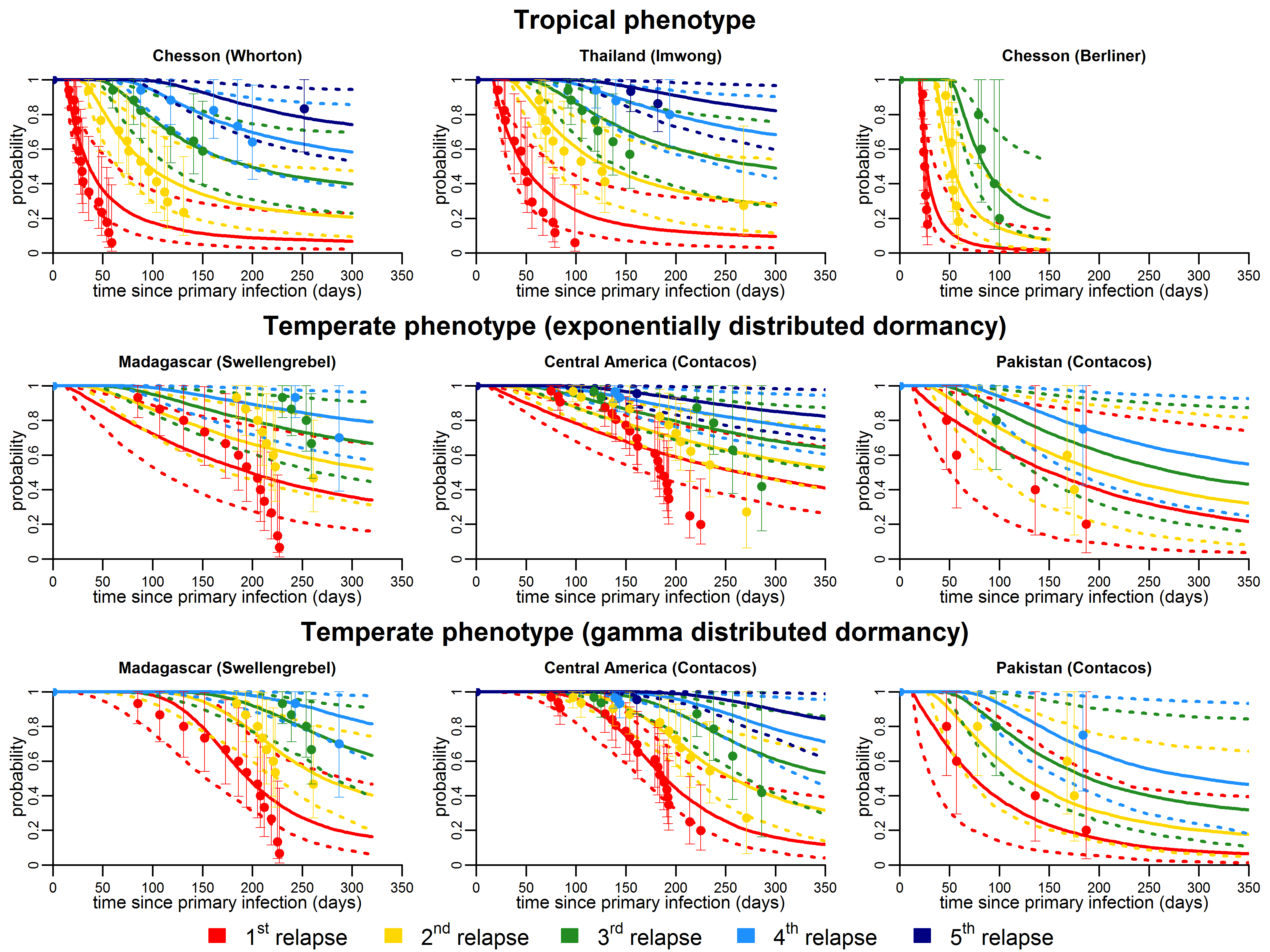


Figure S2: Best-fits of the within-host model to data on time to multiple relapses from six data sets. Round points denote proportion of individuals with ≤ *j* relapses based on survival analysis of the data. Vertical bars denote 95% confidence intervals of the data. Two models were fitted to the temperate datasets: the first with duration of dormancy following an exponential distribution; the second with duration of dormancy following a gamma distribution. The solid curves show the best fit corresponding to the medians of the approximate posterior distributions. The dotted lines represent the 95% credible intervals based on the approximate posterior distributions.

Table S3: Parameter estimates for the within-host hypnozoite density model fitted to data on multiple relapses. Prior and posterior parameter estimates are presented as medians with 95% credible intervals. *N*, ν, 1/µ and 1/α have gamma prior distributions. *d* and σ_d_ have uniform prior distributions. †The 95% confidence interval (95% CI) acceptance criterion resulted in no acceptance in the case of the Central American data for exponentially distributed dormancy.

|  | ***N*** | ***ν*** | **1/µ (days)** | **1/α (days)** | ***d* (days)** | **σ_d_ (days)** | **acceptance** |
| --- | --- | --- | --- | --- | --- | --- | --- |
| Priors | 7.0 (1.3, 50.8) | 2.6 (0.4, 8.0) | 211 (104, 531) | 289 (54, 7900) | U(0,2000) | U(0,1000) |  |
|  |  |  |  |  |  |  |  |
| ***Tropical phenotype*** | | | | | | | |
| Chesson (33) | 5.2 (2.0, 12.6) | 3.0 (0.8, 8.3) | 203 (104, 492) | 376 (105, 1203) | – | – | 0.035 |
| Thailand (34) | 5.4 (1.9, 13.4) | 2.9 (0.8, 8.3) | 213 (108, 526) | 507 (133, 1629) | – | – | 0.039 |
| Chesson (24) | 6.4 (2.8, 14.0) | 3.0 (0.8, 8.4) | 212 (106, 508) | 190 (60, 591) | – | – | 0.009 |
|  |  |  |  |  |  |  |  |
| ***Temperate phenotype (exponentially distributed dormancy)*** | | | | | | | |
| Madagascar (35) | 5.7 (2.2, 12.8) | 2.9 (0.7, 8.3) | 241 (120, 573) | 465 (108, 2312) | 181 (67, 424) | – | 0.0001 |
| Cen. America (37) | 6.8 (2.6, 15.2) | 2.8 (0.6, 8.0) | 245 (127, 598) | 273 (61, 1451) | 293 (116, 631) | – | † |
| Pakistan (36) | 8.3 (3.2, 18.0) | 2.6 (0.5, 7.8) | 227 (114, 567) | 34 (11, 293) | 231 (56, 1201) | – | 0.009 |
|  |  |  |  |  |  |  |  |
| ***Temperate phenotype (gamma distributed dormancy)*** | | | | | | | |
| Madagascar (35) | 6.1 (2.2, 14.2) | 2.9 (0.8, 8.1) | 216 (112, 507) | 275 (57, 1376) | 161 (99, 220) | 41 (5, 92) | 0.002 |
| Cen. America (37) | 6.4 (2.3, 14.8) | 2.9 (0.7, 8.1) | 217 (115, 516) | 230 (56, 1129) | 162 (116, 207) | 49 (11, 94) | 0.003 |
| Pakistan (36) | 8.0 (2.8, 17.9) | 2.4 (0.4, 7.6) | 217 (107, 544) | 246 (55, 1328) | 69 (7, 157) | 67 (10, 98) | 0.010 |

The estimates of the within-host relapse parameters for each of the six cohorts are presented in Table S3. The parameters for the epidemiology of relapses (*h*, *γ_L_*, *f* and *γ_D_*) can be calculated given the within-host parameters using equations (1-4). The number of relapses following infection with a temperate strain of *P. vivax* must be adjusted to account for the proportion of individuals that clear infection before the end of the dormant stage.

$$h=\Gamma_{K}\left(\gamma_{D}\right)N\frac{\alpha}{\alpha+\mu}$$

where $\Gamma_{K}\left(x\right)=\left(\frac{K\delta}{x+K\delta}\right)^{K}$ . *K* is the estimated shape parameter of the Gamma distribution and can be calculated via$K=\left(\frac{d}{\sigma_{d}}\right)^{2}$ . Table S4 presents estimates of the parameters for the epidemiology of relapse for both the cases of a single infectious bite and multiple infectious bites.

For the three studies with data on temperate strains of *P. vivax* (35-37), the estimated durations of dormancy assuming a gamma distribution are plotted in Figure 4c alongside estimates of seasonality. The estimated degree of seasonality in these study sites was estimated from epidemiological studies with longitudinal follow up for *P. vivax*  malaria (50-52)

Table S4: Predicted parameters for the epidemiology of relapses given estimates of the within-host parameters. Larger numbers of bites are assumed to lead to larger numbers of hypnozoites and hence more relapses and faster times to first relapse.

|  | **bites** | ***h*** | **1/*γ_L_* (days)** | **1/*f*(days)** | **1/*γ_D_* (days)** |
| --- | --- | --- | --- | --- | --- |
| ***Tropical phenotype*** | | | | | |
| Chesson (33) | 1 | 1.8 | 241 | 132 | – |
|  | 3.0 | 5.5 | 370 | 68 | – |
| Thailand (34) | 1 | 1.6 | 278 | 174 | – |
|  | 2.9 | 4.6 | 422 | 91 | – |
| Chesson (24) | 1 | 3.4 | 201 | 59 | – |
|  | 3.0 | 10.1 | 301 | 30 | – |
|  |  |  |  |  |  |
| ***Temperate phenotype (exponentially distributed dormancy)*** | | | | | |
| Madagascar (35) | 1 | 1.3 | 302 | 155 | 458 |
|  | 2.9 | 4.5 | 455 | 81 | 690 |
| Cen. America (37) | 1 | 2.0 | 265 | 82 | 503 |
|  | 2.8 | 6.4 | 387 | 43 | 734 |
| Pakistan (36) | 1 | 4.9 | 66 | 9 | 506 |
|  | 2.6 | 14.2 | 92 | 5 | 708 |
| ***Temperate phenotype (gamma distributed dormancy)*** | | | | | |
| Madagascar(35) | 1 | 1.8 | 237 | 88 | 423 |
|  | 2.9 | 6.1 | 354 | 46 | 632 |
| Cen. America (37) | 1 | 2.2 | 223 | 72 | 434 |
|  | 2.9 | 7.0 | 332 | 37 | 645 |
| Pakistan (36) | 1 | 3.3 | 253 | 68 | 477 |
|  | 2.4 | 8.1 | 347 | 39 | 652 |

**2.4. Routine for stochastic simulation of relapse times**

Here we describe the routine for stochastic simulation of the times of observed relapses of a tropical strain of *P. vivax*. The routine for simulation of the times of relapses of temperate strains of *P. vivax* is similar. We assume that each individual is subject to bites from *ν* infectious mosquitoes each inoculating a geometrically distributed number of hypnozoites with mean *N*. The total number of hypnozoites will therefore follow a negative binomial distribution:

$$\eta\sim NB\left(\nu N,\nu\right)$$

For each of the *ƞ* hypnozoites two events are possible: hypnozoite activation or death. The times to events are simulated according to a Gillespie algorithm. The time between event *i*-1 and event *i* is:

$$\tau_{i}\sim Exp\left(\left(\eta-i+1\right)\left(\alpha+\mu\right)\right)\text{ }i=1...\eta$$

And the time to the *i*^th^ event is

$$T_{i}=\sum_{j=1}^{i} \tau_{j}\text{ }i=1...\eta$$

Denote $T=\left\{T_{i}\right\}_{i=1...\eta}$ to be the vector of event times. Events corresponding to hypnozoite death will be unobserved. Each of the *i* events will be a relapse with probability$\frac{\alpha}{\alpha+\mu}$. The vector of events *T* is updated to include only relapses.

$$T=\left\{T_{i}\left|U(0,1)<\frac{\alpha}{\alpha+\mu}\right.\right\}_{i=1...\eta}$$

Where *U*(0,1) is a random draw from a uniform distribution. *ƞ* is updated to be the length of the vector *T*, i.e. the number of relapses.

Not all relapses are observed. Relapses occurring within the censoring period *T_cen_* after primary infection or a previous relapse are assumed to be undetected. First we account for the period of censoring after primary infection

$$T=\left\{T_{i}\left|T_{i}>T_{cen}\right.\right\}_{i=1...\eta}$$

Next we account for the period of censoring after each relapse. This is done by removing relapses that occur within *T_cen_* of a previous relapse

$$\text{for }j\text{ in }1:\eta\text{ }T=\left\{\left.T_{i}\right|T_{i}\leq T_{j}\text{ or }T_{i}>T_{j}+T_{cen}\right\}_{i=1...\eta}$$

Given the above routine for stochastic simulation of relapse times in an individual, we can simulate the relapse times in a cohort of size *P*. For each individual *p* = 1…*P*, we can simulate the times of observed relapses *T^p^*. Given these times, we can calculate the model simulated probability that an individual has experienced ≤ *j* relapses by time *t*:

$$Y_{j}^{t}=1-\frac{\sum_{p=1}^{P} 1\left(t>T_{j}^{p}\right)}{P}$$

**3. Equations for transmission models**

**3.1. Model 1: *P. falciparum***

Humans are assumed to be in one of two states: susceptible (*S_0_*) or infected (*I­_0_*). Mosquitoes are assumed to be in one of two states: susceptible (*S_M_*) or infectious (*I_M_*). In particular, mosquitoes that are infected but not yet sporozoite positive are considered susceptible. The force of infection on humans is given by λ = *mabI_M_*. Parameter definitions and values are provided in Table 1.

$$\begin{matrix} \frac{dS_{0}}{dt}=-\lambda S_{0}+rI_{0} \\ \frac{dI_{0}}{dt}=\lambda S_{0}-rI_{0} \\ \frac{dS_{M}}{dt}=g-acI_{0}\left(e^{-gn}-I_{M}\right)-gS_{M} \\ \frac{dI_{M}}{dt}=acI_{0}\left(e^{-gn}-I_{M}\right)-gI_{M} \end{matrix}$$

The basic reproduction number$R_{0}^{Pf}$ for the system of equations in (S11) is given by (see Section 6.1):

$$R_{0}^{Pf}=\frac{ma^{2}bce^{-gn}}{gr}$$

**3.2. Model 2: *P. vivax* (tropical)**

The mathematical model for *P. falciparum* transmission outlined in equation (S11) can be extended to incorporate relapses characteristic of tropical strains of *P. vivax* through the addition of states for latent hypnozoites (hypnozoites that can relapse at any time). These states are denoted through sub-script *L*.

$$\begin{matrix} \frac{dS_{0}}{dt}=-\lambda S_{0}+rI_{0}+\gamma_{L}S_{L} \\ \frac{dI_{0}}{dt}=-\lambda I_{0}-rI_{0}+\gamma_{L}I_{L} \\ \frac{dS_{L}}{dt}=-\lambda S_{L}+rI_{L}-fS_{L}-\gamma_{L}S_{L} \\ \frac{dI_{L}}{dt}=\lambda\left(S_{0}+I_{0}+S_{L}\right)-rI_{L}+fS_{L}-\gamma_{L}I_{L} \\ \frac{dS_{M}}{dt}=g-ac\left(I_{0}+I_{L}\right)\left(e^{-gn}-I_{M}\right)-gS_{M} \\ \frac{dI_{M}}{dt}=ac\left(I_{0}+I_{L}\right)\left(e^{-gn}-I_{M}\right)-gI_{M} \end{matrix}$$

New infections will cause blood-stage negative individuals (*S*) to become blood-stage positive (*I*), and individuals without liver-stage infections (subscript 0) to move to the latent stage with liver-stage infection (subscript *L*). Note that if an individual in the latent stage is re-infected they will remain in the latent stage. This is a simplification, as reinfection will lead to the build-up of a hypnozoite reservoir in the liver over time (15).

The basic reproduction number$R_{0,L}^{Pv}$ for the system of equations in (S13) is given by (see Section 6.2):

$$R_{0,L}^{Pv}=\frac{ma^{2}bce^{-gn}}{gr}\left(1+\frac{fr}{\gamma_{L}\left(f+r+\gamma_{L}\right)}\right)$$

If there are no relapses (i.e. if *f* = 0) then equation (S14) reduces to equation (S12).

**3.3. Model 3: *P. vivax* (temperate)**

The system of equations describing tropical strains of *P. vivax* can be extended to incorporate the period of dormancy characteristic of temperate strains of *P. vivax*. This can be done through the addition of states representing dormancy denoted below by sub-script *D*.

$$\begin{matrix} \frac{dS_{0}}{dt}=-\lambda S_{0}+rI_{0}+\gamma_{L}S_{L}+\gamma_{D}S_{D} \\ \frac{dI_{0}}{dt}=-\lambda I_{0}-rI_{0}+\gamma_{L}I_{L}+\gamma_{D}I_{D} \\ \frac{dS_{L}}{dt}=-\lambda S_{L}-fS_{L}+rI_{L}-\gamma_{L}S_{L}+\gamma_{D}S_{LD}+\delta S_{D}+\delta S_{LD} \\ \frac{dI_{L}}{dt}=-\lambda I_{L}+fS_{L}-rI_{L}-\gamma_{L}I_{L}+\gamma_{D}I_{LD}+\delta I_{D}+\delta I_{LD} \\ \frac{dS_{D}}{dt}=-\lambda S_{D}+rI_{D}+\gamma_{L}S_{LD}-\gamma_{D}S_{D}-\delta S_{D} \\ \frac{dI_{D}}{dt}=\lambda\left(S_{0}+S_{D}+I_{0}\right)-rI_{D}+\gamma_{L}I_{LD}-\gamma_{D}I_{D}-\delta I_{D} \\ \frac{dS_{LD}}{dt}=-\lambda S_{LD}-fS_{LD}+rI_{LD}-\gamma_{L}S_{LD}-\gamma_{D}S_{LD}-\delta S_{LD} \\ \frac{dI_{LD}}{dt}=\lambda\left(S_{LD}+S_{L}+I_{L}\right)+fS_{LD}-rI_{LD}-\gamma_{L}I_{LD}-\gamma_{D}I_{LD}-\delta I_{LD} \\ \frac{dS_{M}}{dt}=g-ac\left(I_{0}+I_{L}+I_{D}+I_{LD}\right)\left(e^{-gn}-I_{M}\right)-gS_{M} \\ \frac{dI_{M}}{dt}=ac\left(I_{0}+I_{L}+I_{D}+I_{LD}\right)\left(e^{-gn}-I_{M}\right)-gI_{M} \end{matrix}$$

In equation (S15) it is assumed that the duration of dormancy is exponentially distributed. The basic reproduction number$R_{0,D}^{Pv}$for the system is:

$$R_{0,D}^{Pv}=\frac{ma^{2}bce^{-gn}}{g}\frac{1}{r}\left(1+\frac{\delta fr\left(\delta+r+\gamma_{L}+\gamma_{D}\right)}{\gamma_{L}\left(f+r+\gamma_{L}\right)\left(\delta+\gamma_{D}\right)\left(\delta+r+\gamma_{D}\right)}\right)$$

As the duration of long-latency approaches zero, equation (S16) simplifies to equation (S14), i.e. as$d=1/\delta\to0$ ,$R_{0,D}^{Pv}\to R_{0,L}^{Pv}$.

In the model described in equation (S15) the period of dormancy is described by an exponential distribution. We can instead choose to model this as a Gamma distribution (28). In the system of equations below, the Gamma distribution is assumed to be the sum of *K* exponential distributions, each with rate *K*δ.

$$\begin{matrix} \frac{dS_{0}}{dt}=-\lambda S_{0}+rI_{0}+\gamma_{L}S_{L}+\gamma_{D}\sum_{j=1}^{K} S_{D}^{j} \\ \frac{dI_{0}}{dt}=-\lambda I_{0}-rI_{0}+\gamma_{L}I_{L}+\gamma_{D}\sum_{j=1}^{K} I_{D}^{j} \\ \frac{dS_{L}}{dt}=-\lambda S_{L}-fS_{L}+rI_{L}-\gamma_{L}S_{L}+\gamma_{D}\sum_{j=1}^{K} S_{LD}^{j}+K\delta S_{D}^{K}+K\delta S_{LD}^{K} \\ \frac{dI_{L}}{dt}=-\lambda I_{L}+fS_{L}-rI_{L}-\gamma_{L}I_{L}+\gamma_{D}\sum_{j=1}^{K} I_{LD}^{j}+K\delta I_{D}^{K}+K\delta I_{LD}^{K} \\ \frac{dS_{D}^{1}}{dt}=-\lambda S_{D}^{1}+rI_{D}^{1}+\gamma_{L}S_{LD}^{1}-\gamma_{D}S_{D}^{1}-K\delta S_{D}^{1} \\ \frac{dS_{D}^{k}}{dt}=-\lambda S_{D}^{k}+rI_{D}^{k}+\gamma_{L}S_{LD}^{k}-\gamma_{D}S_{D}^{k}+K\delta S_{D}^{k-1}-K\delta S_{D}^{k} \\ \frac{dI_{D}^{1}}{dt}=\lambda\left(S_{0}+I_{0}\right)+\lambda S_{D}^{1}-rI_{D}^{1}+\gamma_{L}I_{LD}^{1}-\gamma_{D}I_{D}^{1}-K\delta I_{D}^{1} \\ \frac{dI_{D}^{k}}{dt}=\lambda S_{D}^{k}-rI_{D}^{k}+\gamma_{L}I_{LD}^{k}-\gamma_{D}I_{D}^{k}+K\delta I_{D}^{k-1}-K\delta I_{D}^{k} \\ \frac{dS_{LD}^{1}}{dt}=-\lambda S_{LD}^{1}-fS_{LD}^{1}+rI_{LD}^{1}-\gamma_{L}S_{LD}^{1}-\gamma_{D}S_{LD}^{1}-K\delta S_{LD}^{1} \\ \frac{dS_{LD}^{k}}{dt}=-\lambda S_{LD}^{k}-fS_{LD}^{k}+rI_{LD}^{k}-\gamma_{L}S_{LD}^{k}-\gamma_{D}S_{LD}^{k}+K\delta S_{LD}^{k-1}-K\delta S_{LD}^{k} \\ \frac{dI_{LD}^{1}}{dt}=\lambda\left(S_{L}+I_{L}\right)+\lambda S_{LD}^{1}+fS_{LD}^{1}-rI_{LD}^{1}-\gamma_{L}I_{LD}^{1}-\gamma_{D}I_{LD}^{1}-K\delta I_{LD}^{1} \\ \frac{dI_{LD}^{k}}{dt}=\lambda S_{LD}^{k}+fS_{LD}^{k}-rI_{LD}^{k}-\gamma_{L}I_{LD}^{k}-\gamma_{D}I_{LD}^{k}+K\delta I_{LD}^{k-1}-K\delta I_{LD}^{k} \\ \frac{dS_{M}}{dt}=g-ac\left(\sum_{j=1}^{K} \left(I_{0}^{j}+I_{L}^{j}+I_{D}^{j}+I_{LD}^{j}\right)\right)\left(e^{-gn}-I_{M}\right)-gS_{M} \\ \frac{dI_{M}}{dt}=ac\left(\sum_{j=1}^{K} \left(I_{0}^{j}+I_{L}^{j}+I_{D}^{j}+I_{LD}^{j}\right)\right)\left(e^{-gn}-I_{M}\right)-gI_{M} \end{matrix}$$

where the super-script k takes values in the range 2:*K*. Denote $R_{0,D_{K}}^{Pv}$to be the basic reproduction number for the above system of equations. It is possible to derive an analytic expression for$R_{0,D_{k}}^{Pv}$, but it is not algebraically convenient (see Section 6.4). It can instead be calculated numerically using the routine described by van den Driessche and Watmough (53).

**3.4. Transmission dynamics**

Figure S3 shows a comparison of the model predicted transmission dynamics for *P. falciparum* (no relapses), tropical strains of *P. vivax* (latent hypnozoites but no dormant hypnozoites), and temperate strains of *P. vivax* with different distributions for the duration of dormancy.

**
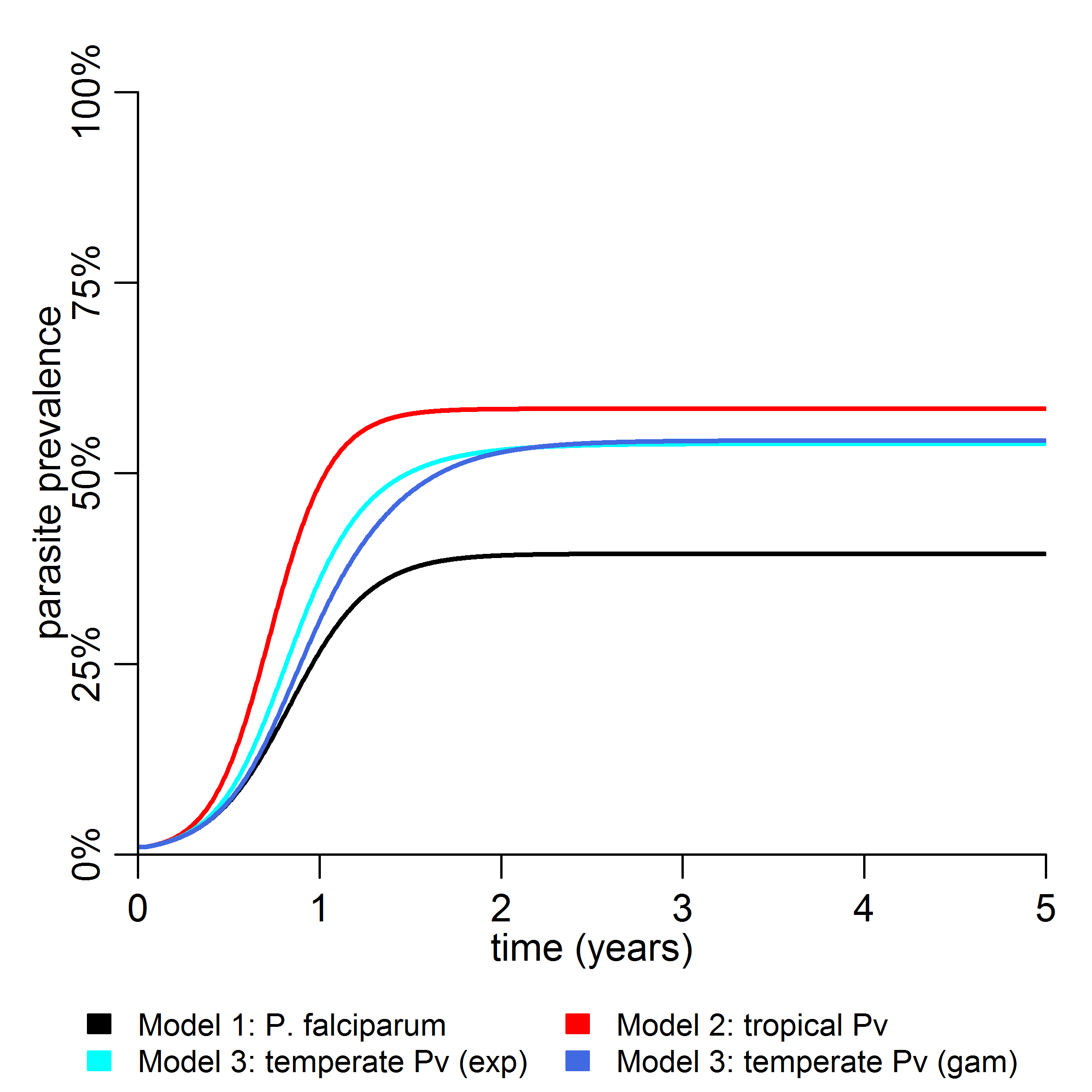
**

Figure S3: Changes in parasite prevalence (assumed to begin at 1%) predicted by mathematical models. For the model of temperate strains of *P. vivax*, the duration of dormancy is assumed to follow either an exponential or gamma distribution. It is assumed that there are *m* = 2 mosquitoes per human.

**4. Seasonality of malaria transmission**

The seasonality of malaria transmission in a given environmental niche will be driven by temperature and rainfall-dependent fluctuations in mosquito density and vectorial capacity (1). We model seasonality by assuming that mosquito population densities follow a periodic pattern (54). We ignore variation in factors such as the duration of sporogony and mosquito life expectancy due to seasonal variation in temperature. Following the approach of Griffin (29) we assume that seasonal mosquito densities are given by

where time *t* is in days. m_0_ is the average annual mosquito density. ε = 0.001 is the ratio between mosquito density at the peak of the season and the trough of the season. *κ* is a parameter determining the seasonality – larger values of *κ* correspond to higher degrees of seasonality. *B* is the Beta function which enters the expression to ensure that the function is normalised so that the average of *m*(*t*) over a year is *m_0_*.

Figure S4a shows how the seasonality parameter *κ* affects the proportion of exposure to mosquitoes occurring in the peak three months. FigureS4b shows examples of several seasonal profiles. Examples of the transmission dynamics of a temperate strain of *P. vivax* are shown for a low seasonality setting (Figure S4c) and a high seasonality setting (Figure S4d).


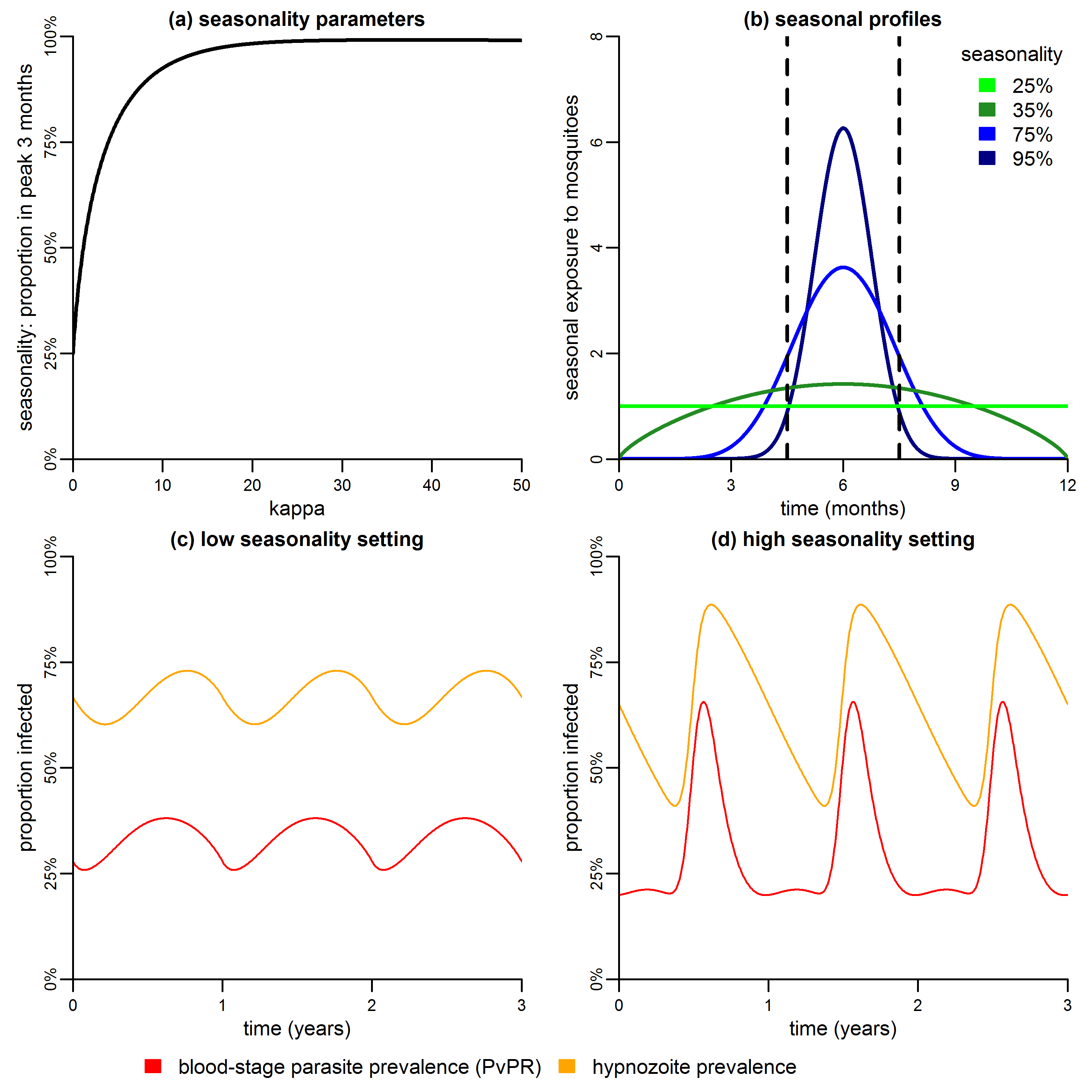


Figure S4: (a) Relationship between the seasonality parameter *κ* and the proportion of exposure to mosquitoes in the peak three months of the year. (b) Seasonal profiles of mosquito density used to drive transmission. The curves are given by equation (S18). The vertical dashed lines denote the three months where transmission is at its highest. In the most extreme case shown the peak three months account for 95% of exposure to mosquitoes. (c) Example of *P. vivax* transmission dynamics (temperate strain with duration of dormancy following a gamma distribution)) in a low seasonality setting, where 35% of transmission is assumed to occur in the peak 3 months, corresponding to κ = 0.37. (d) Example of *P. vivax* transmission dynamics (temperate strain) in a high seasonality setting, where 95% of transmission is assumed to occur in the peak 3 months, corresponding to κ = 12.3.

**5. Optimisation of *R_0_***

**5.1. *P. vivax* (tropical) in a non-seasonal setting**

The basic reproduction number for tropical strains of *P. vivax* is

$$R_{0,L}^{Pv}=\frac{ma^{2}bce^{-gn}}{gr}\left(1+\frac{fr}{\gamma_{L}\left(f+r+\gamma_{L}\right)}\right)$$

The parameters describing the epidemiology of relapses *f* and *γ_L_* can be expressed in terms of the within-host parameters α, µ and *N* and inserted into equation (S19) to give:

$$R_{0,L}^{Pv}=\frac{ma^{2}bce^{-gn}}{gr}\left(1+\frac{\alpha r}{\mu+\alpha}\frac{N\log\left(N+1\right)}{\left(N+1\right)\alpha+\mu+r\log\left(N+1\right)}\right)$$

We can optimise$R_{0,L}^{Pv}$ with respect to α by differentiating and setting equal to zero.

Finally, the relapse frequency that maximises$R_{0,L}^{Pv}$ is

$$f_{\text{max}}=\frac{N}{\log(N+1)}\sqrt{\frac{\mu r\log(N+1)+\mu^{2}}{N+1}}$$

**5.2. *P. vivax* (temperate) in a seasonal setting**

Figure 4c shows how the time to first relapse is predicted to vary with the degree of seasonality. This was done by calculating the duration of dormancy *d* that optimises the basic reproduction number *R_0_* for each seasonal profile. Figure S5 shows how *R_0_* varies across the full parameter space of seasonality and time to first relapse using a heatmap.


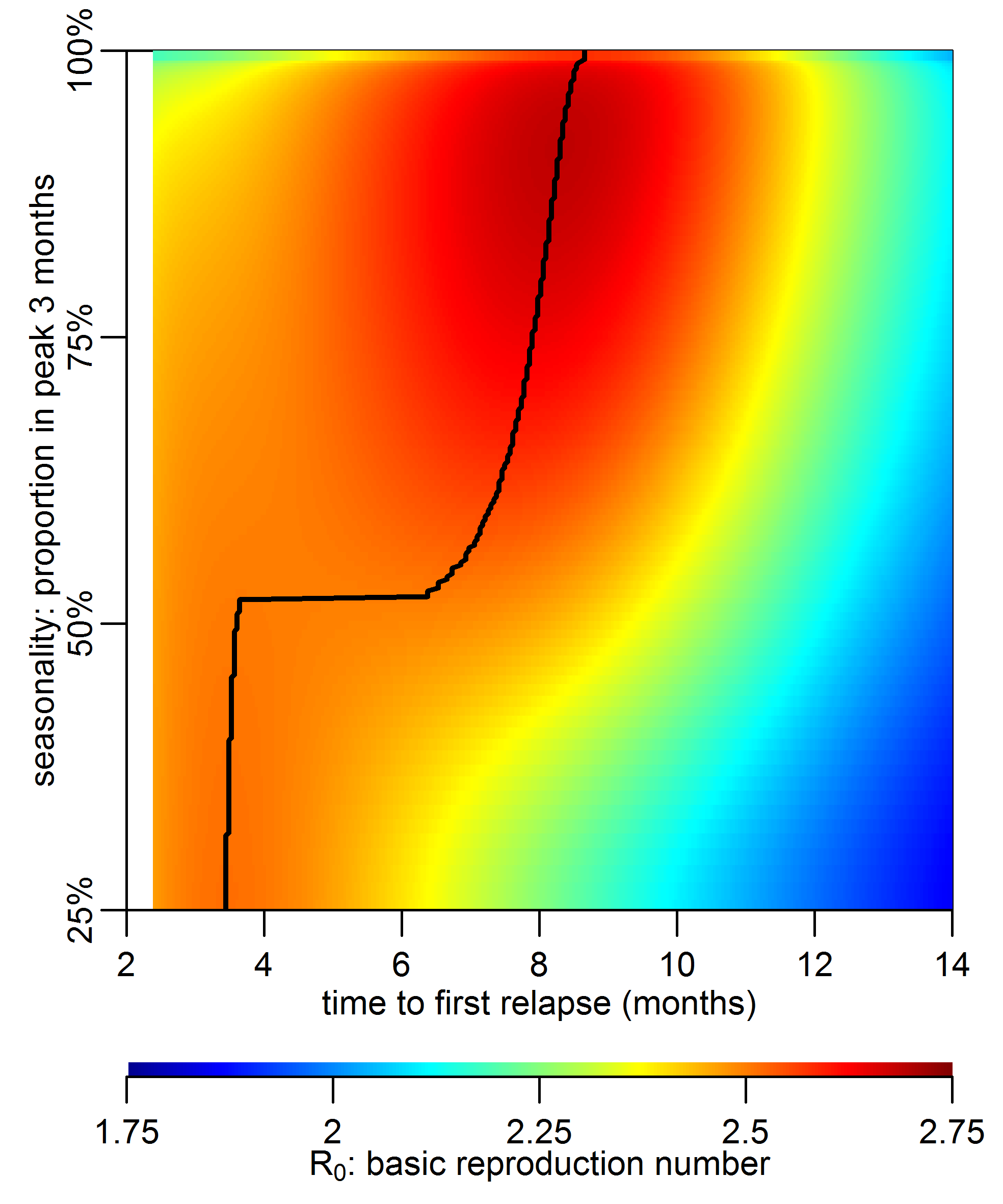


Figure S5: Heatmap showing how the basic reproduction number for temperate strains of *P. vivax* is predicted to vary with time to first relapse and the seasonality. The time to first relapse is comprised of expected time in the dormant stage (*d*) plus expected time in the latent stage (1/*f*). The degree of seasonality is measured as the proportion of exposure to mosquitoes occurring in the peak three months.

**6. Calculation of basic reproduction number *R_0_***

**6.1. Model 1: *P. falciparum***

The basic reproduction number for *P. falciparum* was first defined by MacDonald (25). Smith *et al* (27) present an intuitive heuristic approach for calculating$R_{0}^{Pf}$by tracking the parasite’s progression through an entire lifecycle and calculating the expected number of secondary infections arising from a primary infection (Figure S6). An infected human will have blood-stage parasites for 1/*r* days (note that this assumes that infected humans are always infectious and ignores gametocyte dynamics (55)). Every day they will be bitten *ma* times. Each of these bites will result in transmission to a mosquito with probability *c*. The probability that an infected mosquito will survive the *n* days of sporogony to allow transmission to the next person is *e*^-^*^gn^*. The number of bites that an infected mosquito will take on humans is *a*/*g* and each of these bites will result in transmission with probability *b*. Multiplying all of these factors together gives the basic reproduction number for *P. falciparum*:

$$R_{0}^{Pf}=\frac{ma^{2}bce^{-gn}}{gr}$$

A key property of equation (S24) is that *R_0_^Pf^* is directly proportional to the time spent with blood-stage parasites (*T_BS_*). For the model of *P. falciparum* considered here *T_BS_* = 1/*r*.


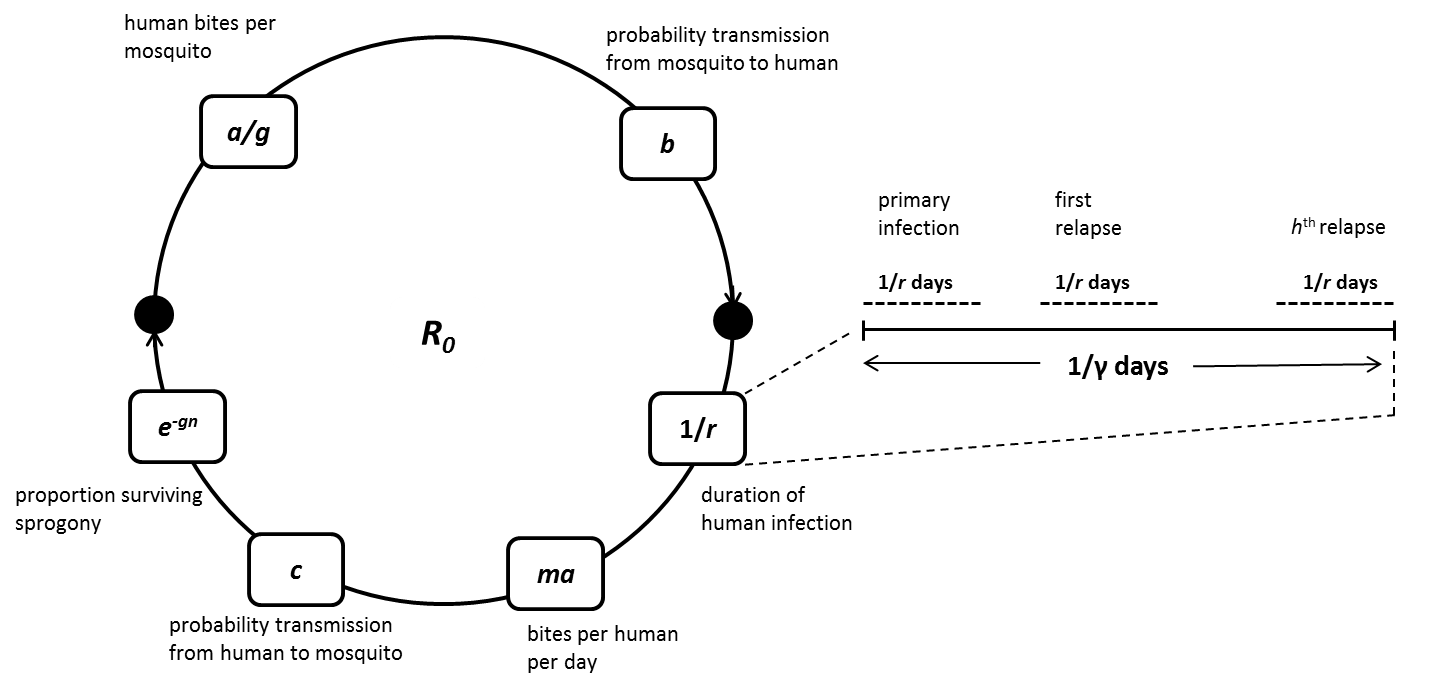


Figure S6: The life cycle of malaria transmission and *R_o_* (adapted from Smith *et al* (27)). *R_0_* can be calculated by multiplying each factor in the lifecycle. For the case of *P. falciparum* the duration of blood-stage infection in a human is *T_BS_* = 1/*r*. For the case of *P. vivax* the duration of blood-stage infection must be expanded to account for the additional time spent with blood-stage parasites arising from relapses.

**6.2. Model 2: *P. vivax* (tropical)**

Similarly to *P. falciparum*, calculation of the basic reproduction number of *P. vivax* malaria will depend on the underlying model assumptions. It has been previously estimated for other *P. vivax* transmission models (56-59). For the model structure utilised here, the basic reproduction number for tropical strains of *P. vivax*$R_{0,L}^{Pv}$can be calculated using an extension of the heuristic approach described by Smith *et al* (27) (Figure S6). The key difference for *P. vivax* is that relapse infections will alter the time an infected individual is capable of transmitting to mosquitoes. For tropical strains of *P. vivax*, *T_BS,L_* can be calculated by considering the transitions between states depicted in Figure S7.


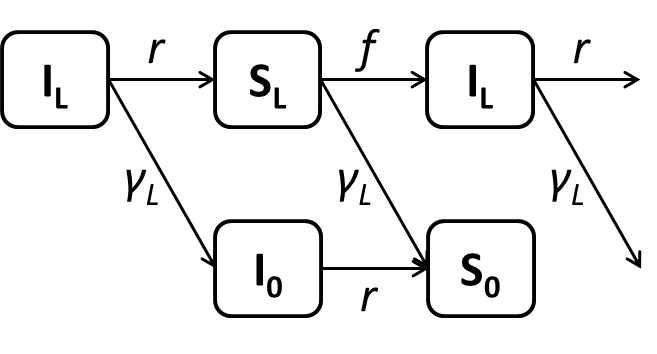


Figure S7: Transition between states for an individual infected with both blood-stages and latent liver-stages of a tropical strain of *P. vivax*.

The time spent with blood-stage parasites *T*_BS,L_ can be calculated as follows:

$$\begin{matrix} T_{BS,L}=\frac{1}{r+\gamma_{L}}+\frac{\gamma_{L}}{r+\gamma_{L}}\frac{1}{r}+\frac{r}{r+\gamma_{L}}\frac{f}{f+\gamma_{L}}T_{BS,L} \\ T_{BS,L}=\frac{1}{r}\left(1+\frac{fr}{\gamma_{L}\left(f+r+\gamma_{L}\right)}\right) \end{matrix}$$

And hence the basic reproduction number can be calculated as:

$$R_{0,L}^{Pv}=\frac{ma^{2}bce^{-gn}}{g}T_{BS,L}=\frac{ma^{2}bce^{-gn}}{gr}\left(1+\frac{fr}{\gamma_{L}\left(f+r+\gamma_{L}\right)}\right)$$

**6.3. Model 3: *P. vivax* (temperate with exponential dormancy)**

The expected time spent with blood-stage parasites following infection with a temperate strain of *P. vivax* (exponential dormancy) can be calculated by considering the transitions between states depicted in Figure S8.


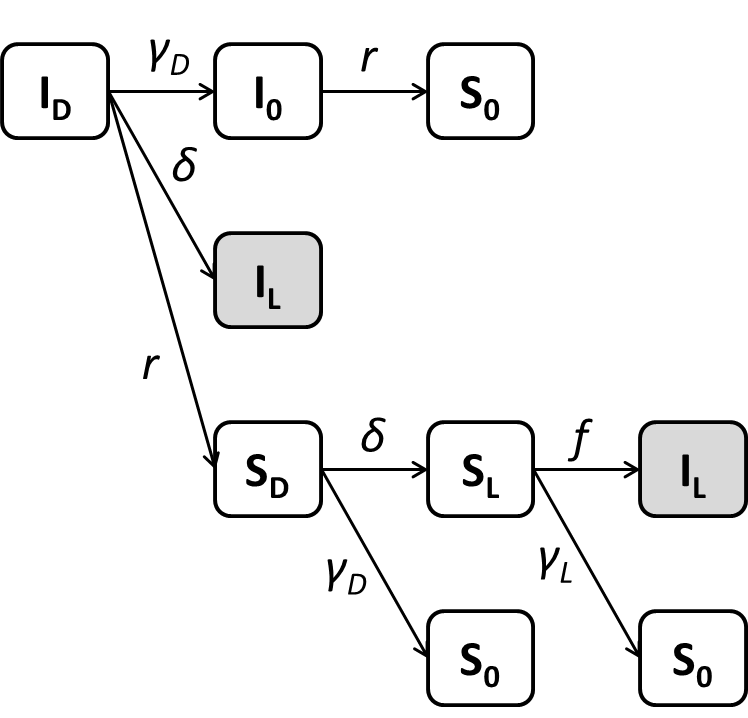


Figure S8: Transition between states for an individual infected with both blood-stages and liver-stages of a tropical strain of *P. vivax*. Once at the *I_L_* states (shaded grey), the time spent with blood-stage parasites is the same as in the model for the tropical strain of *P. vivax* (equation (S25)).

The expected time with blood-stage parasites can be calculated from the flow-diagram in Figure S8 as follows:

$$\begin{matrix} T_{BS,D}=\frac{1}{\gamma_{D}+\delta+r}+\frac{\gamma_{D}}{\gamma_{D}+\delta+r}\frac{1}{r}+\frac{\delta}{\gamma_{D}+\delta+r}T_{BS,L}+\frac{r}{\gamma_{D}+\delta+r}\frac{\delta}{\gamma_{D}+\delta}\frac{f}{\gamma_{L}+f}T_{BS,\mathrm{L}} \\ T_{BS,D}=\frac{1}{r}\left(1+\frac{\delta fr\left(d+r+\gamma_{L}+\gamma_{D}\right)}{\gamma_{L}\left(f+r+\gamma_{L}\right)\left(\delta+\gamma_{D}\right)\left(\delta+r+\gamma_{D}\right)}\right) \end{matrix}$$

And hence the basic reproduction number is:

$$R_{0,D}^{Pv}=\frac{ma^{2}bce^{-gn}}{g}\frac{1}{r}\left(1+\frac{\delta fr\left(\delta+r+\gamma_{L}+\gamma_{D}\right)}{\gamma_{L}\left(f+r+\gamma_{L}\right)\left(\delta+\gamma_{D}\right)\left(\delta+r+\gamma_{D}\right)}\right)$$

**6.4. Calculation of *R_0_* using the method of van den Driessche and Watmough**

**Model 1: *P. falciparum***

An alternative approach utilises the methodology of van den Driessche and Watmough (53) which allows the basic reproduction number to be calculated from a system of differential equations. Equation (S11) can be rewritten in the form$\frac{d\mathbf{x}}{dt}=\Lambda\left(\mathbf{x}\right)=F\left(\mathbf{x}\right)-V\left(\mathbf{x}\right)$ as follows:

The disease free equilibrium is$x_{0}=\left(0,0,1,1\right)$. The 2 x 2 matrices *F* and *V* can be calculated from equation (S29) as follows:

$$F=\left[\frac{\partial F_{i}}{\partial x_{j}}\left(x_{0}\right)\right]\text{ and }V=\left[\frac{\partial V_{i}}{\partial x_{j}}\left(x_{0}\right)\right]\text{ with }1\leq i,j\leq2$$

Evaluating *F* and *V* from equation (S30) gives:

$$F=\left(\begin{matrix} 0 & mab \\ ace^{-gn} & 0 \end{matrix}\right)\text{ }V=\left(\begin{matrix} r & 0 \\ 0 & g \end{matrix}\right)$$

$R_{0}^{Pf}$can be calculated as the spectral radius of the matrix *FV*^-1^ as follows:

$$R_{0}^{Pf}=\rho\left(FV^{-1}\right)=\frac{ma^{2}bce^{-gn}}{gr}$$

**Model 2: *P. vivax* (tropical)**

Equation (S13) can be rewritten in the form$\frac{d\mathbf{x}}{dt}=\Lambda\left(\mathbf{x}\right)=F\left(\mathbf{x}\right)-V\left(\mathbf{x}\right)$ as follows:

The disease free equilibrium is$x_{0}=\left(0,0,0,0,1,1\right)$. The 4 x 4 matrices *F* and *V* can be calculated from equation (S33) as follows:

$$F=\left[\frac{\partial F_{i}}{\partial x_{j}}\left(x_{0}\right)\right]\text{ and }V=\left[\frac{\partial V_{i}}{\partial x_{j}}\left(x_{0}\right)\right]\text{ with }1\leq i,j\leq4$$

Evaluating *F* and *V* from equation (S34) gives:

$$F=\left(\begin{matrix} 0 & 0 & 0 & 0 \\ 0 & 0 & 0 & 0 \\ 0 & 0 & 0 & mab \\ ace^{-gn} & 0 & ace^{-gn} & 0 \end{matrix}\right)\text{ }V=\left(\begin{matrix} r & 0 & -\gamma_{L} & 0 \\ 0 & f+\gamma_{L} & -r & 0 \\ 0 & -f & \gamma_{L}+r & 0 \\ 0 & 0 & 0 & g \end{matrix}\right)$$

$R_{0,L}^{Pv}$can be calculated as the spectral radius of the matrix *FV*^-1^ as follows:

$$R_{0,L}^{Pv}=\rho\left(FV^{-1}\right)=\frac{ma^{2}bce^{-gn}}{gr}\left(1+\frac{fr}{\gamma_{L}\left(f+r+\gamma_{L}\right)}\right)$$

**Model 3: *P. vivax* (temperate with exponentially distributed dormancy)**

Equation (S15) can be rewritten in the form$\frac{d\mathbf{x}}{dt}=\Lambda\left(\mathbf{x}\right)=F\left(\mathbf{x}\right)-V\left(\mathbf{x}\right)$ as follows:

The disease free equilibrium is$x_{0}=\left(0,0,0,0,0,0,0,0,1,1\right)$. The 8 x 8 matrices *F* and *V* can be calculated from equation (S37) as follows:

$$F=\left(\begin{matrix} 0 & 0 & 0 & 0 & 0 & 0 & 0 & 0 \\ 0 & 0 & 0 & 0 & 0 & 0 & 0 & 0 \\ 0 & 0 & 0 & 0 & 0 & 0 & 0 & 0 \\ 0 & 0 & 0 & 0 & 0 & 0 & 0 & 0 \\ 0 & 0 & 0 & 0 & 0 & 0 & 0 & mab \\ 0 & 0 & 0 & 0 & 0 & 0 & 0 & 0 \\ 0 & 0 & 0 & 0 & 0 & 0 & 0 & 0 \\ ace^{-gn} & 0 & ace^{-gn} & 0 & ace^{-gn} & 0 & ace^{-gn} & 0 \end{matrix}\right)$$

$$V=\left(\begin{matrix} r & 0 & -\gamma_{L} & 0 & -\gamma_{D} & 0 & 0 & 0 \\ 0 & f+\gamma_{L} & -r & -\delta& 0 & -\gamma_{D}-\delta& 0 & 0 \\ 0 & -f & r+\gamma_{L} & 0 & -d & 0 & -\gamma_{D}-\delta& 0 \\ 0 & 0 & 0 & \gamma_{D}+\delta& -r & -\gamma_{L} & 0 & 0 \\ 0 & 0 & 0 & 0 & r+\gamma_{D}+\delta& 0 & -\gamma_{L} & 0 \\ 0 & 0 & 0 & 0 & 0 & f+\gamma_{L}+\gamma_{D}+\delta& -r & 0 \\ 0 & 0 & 0 & 0 & 0 & -f & r+\gamma_{L}+\gamma_{D}+\delta& 0 \\ 0 & 0 & 0 & 0 & 0 & 0 & 0 & g \end{matrix}\right)$$

$R_{0,D}^{Pv}$ can be calculated as the spectral radius of the matrix *FV*^-1^ as follows:

$$R_{0,D}^{Pv}=\rho\left(FV^{-1}\right)=\frac{ma^{2}bce^{-gn}}{g}\frac{1}{r}\left(1+\frac{\delta fr\left(\delta+r+\gamma_{L}+\gamma_{D}\right)}{\gamma_{L}\left(f+r+\gamma_{L}\right)\left(\delta+\gamma_{D}\right)\left(\delta+r+\gamma_{D}\right)}\right)$$

**Model 3: *P. vivax* (temperate with gamma distributed dormancy)**

The methods applied above can also be used for the case of a *P. vivax* transmission model where the duration of dormancy follows a gamma distribution. This requires the construction of (4+4K)*(4+4K) matrices. Analytic derivations of the dominant eigenvalue are particularly challenging and hence the method is implemented numerically.

**6.5. Calculation of *R_0_* in seasonal settings using Floquet theory**

In the derivation of *R_0_* above it is assumed that $\Lambda\left(\mathbf{x}\right)$ does not vary with time. In the periodic seasonal settings considered here, transmission will vary due to seasonality in mosquito populations such that$\Lambda\left(\mathbf{x},t\right)=\Lambda\left(\mathbf{x},t+T\right)$, where *T* = 365 days is the period of the seasonal cycle. Here we use Floquet theory (29, 30), a branch of applied mathematics dealing with periodic differential equations, to calculate the basic reproduction number in seasonal settings. Denote$\mathbf{\Lambda}(t)$ to be the linearised square matrix about the disease-free equilibrium $\mathbf{x}_{0}$such that

$$\mathbf{\Lambda}_{ij}(t)=\left[\frac{\partial\Lambda_{i}(t)}{\partial x_{j}}\left(\mathbf{x}_{0}\right)\right]$$

The linearised version of the model has the form:

$$\frac{d\mathbf{x}(t)}{dt}=\mathbf{\Lambda}(t)\mathbf{x}(t)$$

The monodromy matrix $\mathbf{M}$ of the system in equation (S41) has the property that the disease-free equilibrium $\mathbf{x}_{0}$ is stable if and only if the largest eigenvalue of $\mathbf{M}$ is greater than one (30). $\mathbf{M}$ can be calculated by setting the initial condition of equation (S41),$\mathbf{x}(t=0)$, to the identity matrix and numerically simulating over one period so that$\mathbf{M}=\mathbf{x}\left(t=T\right)$.

This method can be used to calculate *R_o_* as follows (29). If the mosquito density *m*(*t*) is multiplied by a factor *ξ*, then *R_0_* is also multiplied by *ξ*. In equation (S41), if we replace *m*(*t*) with *ξm*(*t*), we can find the value of *ξ* that ensures that the largest eigenvalue of$\mathbf{M}$ is one. Denote this value as *ξ_0_*. The basic reproduction number is then *R_0_* = 1/ *ξ_0_*. In practice, calculation of *ξ_0_* can be done using a root-finding algorithm (e.g. by interval bisection).

**7. Multi-strain models**

Gog and Grenfell (31) describe how an SIR model for an influenza-like illness can be extended to incorporate strain dynamics and competition between strains. The dynamics of a single strain of influenza are described by equation (S42).

$$\begin{matrix} \frac{dS}{dt}=\mu-\beta SI-\mu S \\ \frac{dI}{dt}=\beta SI-\nu I-\mu I \end{matrix}$$

Equation (S42) can be extended to account for multiple strains (indexed by *i*) as follows:

$$\begin{matrix} \frac{dS^{i}}{dt}=\mu-\sum_{j} \beta^{j}S^{i}\psi_{ij}I^{j}-\mu S^{i} \\ \frac{dI^{i}}{dt}=\beta^{i}S^{i}I^{i}-\nu^{i}I^{i}-\mu I^{i} \end{matrix}$$

where the matrix ψ_ij_ determines the degree of cross-strain immunity. An important feature of this system of equations is that co-infections are not explicitly modelled. For example for a 2-strain model we would have *S*^1^ + *I*^1^ = 1 and *S*^2^ + *I*^2^ = 1. Thus we know the prevalence of each strain but not the prevalence of co-infection. Tracking co-infection would require modelling the states *S*^1^*S*^2^, *I*^1^*S*^2^, *S*^1^*I*^2^ and *I*^1^*I*^2^, where *S*^1^*S*^2^ + *I*^1^*S*^2^ + *S*^1^*I*^2^ + *I*^1^*I*^2^ = 1.

In a similar manner to equation (S43) a Ross-MacDonald model of *P. falciparum* malaria as described in equation (S11) can be extended to account for multiple strains and competition between strains as follows:

$$\begin{matrix} \frac{dS_{0}^{i}}{dt}=-\lambda^{i}S_{0}^{i}e^{-\sum_{j} \psi_{ij}I_{0}^{j}}+rI_{0}^{i} \\ \frac{dI_{0}^{i}}{dt}=\lambda^{i}S_{0}^{i}e^{-\sum_{j} \psi_{ij}I_{0}^{j}}-rI_{0}^{i} \\ \frac{dS_{M}^{i}}{dt}=g-ac\left(\sum_{j} \omega_{ij}I_{0}^{j}\right)\left(e^{-gn}-I_{M}^{i}\right)-gS_{M}^{i} \\ \frac{dI_{M}^{i}}{dt}=ac\left(\sum_{j} \omega_{ij}I_{0}^{j}\right)\left(e^{-gn}-I_{M}^{i}\right)-gI_{M}^{i} \end{matrix}$$

Here the term$e^{-\sum_{j} \psi_{ij}I_{0}^{j}}$ accounts for cross-strain blood-stage immunity. The term$\sum_{j} \omega_{ij}I_{0}^{j}$ accounts for between generation mutation between strains. We assume that cross-strain immunity acts to reduce the probability that an infected individual acquires multiple strains. The cross-strain immunity matrix is defined as follows

where *I* is the number of circulating strains. Figure S9a shows how cross-strain immunity as described by the matrix in equation (S45) with *ψ_0_* = 1 reduces the probability of acquiring new blood-stage infections.

The model also allows for de novo evolution of phenotypes of interest due to random mutation. For example, in the case of temperate strains of *P. vivax*, strains will differ in terms of the duration of dormancy *d^i^*. We assume that mutation can occur once per generation during the mosquito stage, and that the change will follow a Normal distribution with standard deviation σ_ω_. We do not consider potential sexual recombination between multiple strains in a mosquito. The mutation matrix can be defined as follows:

$$\omega_{ij}=e^{-\frac{\left(d^{i}-d^{j}\right)^{2}}{2\sigma_{\omega}^{2}}}/\sum_{h=1}^{I} e^{-\frac{\left(d^{i}-d^{h}\right)^{2}}{2\sigma_{\omega}^{2}}}$$

Figure S9b shows an example of the distribution in change of duration of dormancy from generation to generation. In the simulations presented in Figure 6 of the manuscript, we ignore evolution due to random mutation and hence σ_ω_ = 0.

*
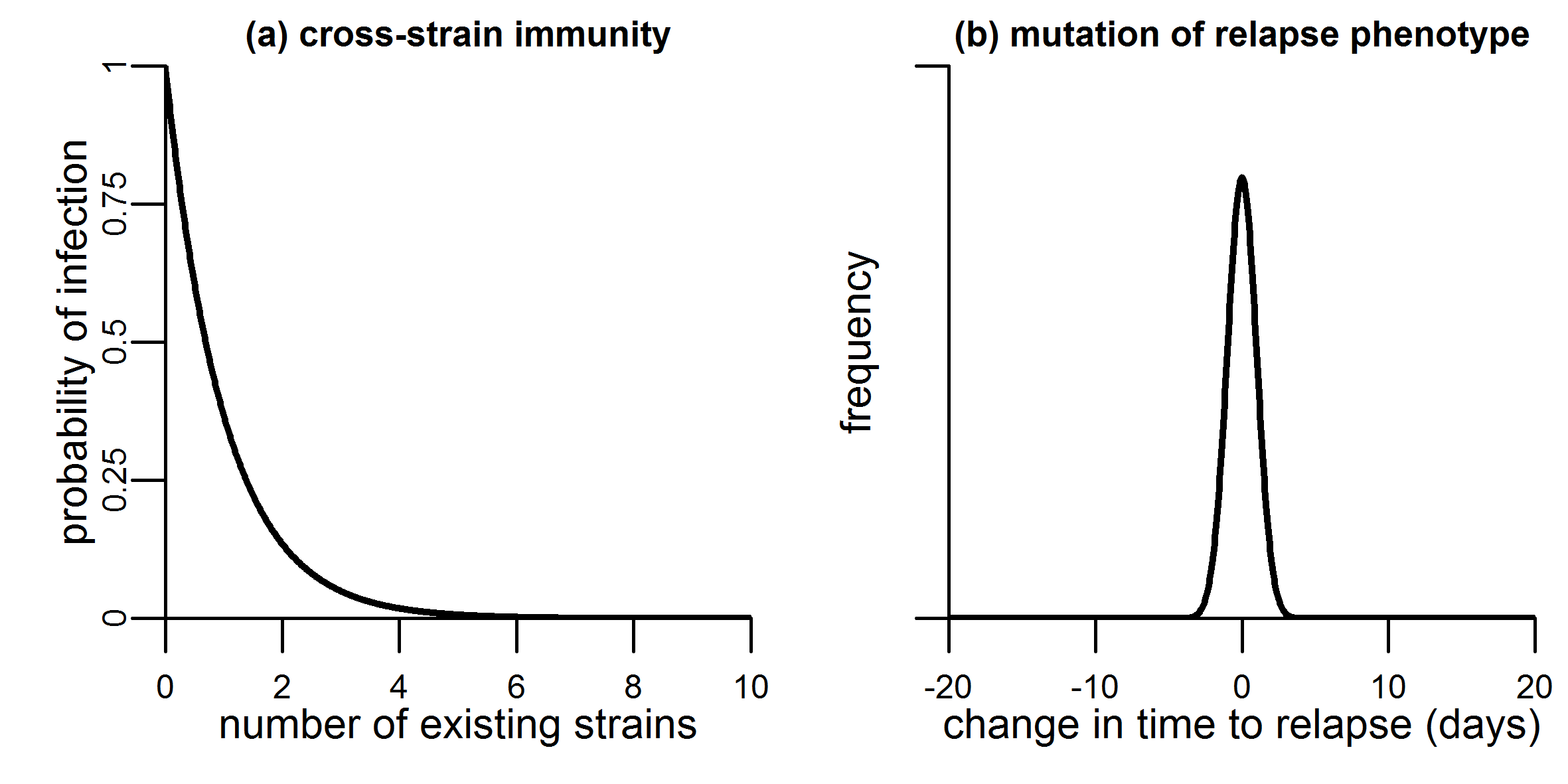
*

Figure S9: (a) An individual infected with multiple strains of *P. vivax* is assumed to have a degree of cross-strain immunity making super-infection less likely. The relationship shown depicts how the probability of acquiring a new infection is reduced if the individual is already infected with other strains. (b) Distribution of change in relapse frequency from generation to generation due to de novo evolution by random mutation.

The model for tropical strains of *P. vivax* described in equation (S13) can also be extended to account for multiple strains:

$$\begin{matrix} \frac{dS_{0}^{i}}{dt}=-\lambda^{i}S_{0}^{i}+rI_{0}^{i}+\gamma_{L}^{i}S_{L}^{i} \\ \frac{dI_{0}^{i}}{dt}=-\lambda^{i}I_{0}^{i}-rI_{0}^{i}+\gamma_{L}^{i}I_{L}^{i} \\ \frac{dS_{L}^{i}}{dt}=\lambda^{i}S_{0}^{i}\left(1-e^{-\sum_{j} \psi_{ij}\left(I_{0}^{j}+I_{L}^{j}\right)}\right)-\left(\lambda^{i}S_{L}^{i}+f^{i}S_{L}^{i}\right)e^{-\sum_{j} \psi_{ij}\left(I_{0}^{j}+I_{L}^{j}\right)}+rI_{L}^{i}-\gamma_{L}^{i}S_{L}^{i} \\ \frac{dI_{L}^{i}}{dt}=\left(\lambda^{i}S_{0}^{i}+\lambda^{i}S_{L}^{i}+f^{i}S_{L}^{i}\right)e^{-\sum_{j} \psi_{ij}\left(I_{0}^{j}+I_{L}^{j}\right)}+\lambda^{i}I_{0}^{i}-rI_{L}^{i}-\gamma_{L}^{i}I_{L}^{i} \\ \frac{dS_{M}^{i}}{dt}=g-ac\left(\sum_{j} \omega_{ij}\left(I_{0}^{j}+I_{L}^{j}\right)\right)\left(I_{0}^{i}+I_{L}^{i}\right)\left(e^{-gn}-I_{M}^{i}\right)-gS_{M}^{i} \\ \frac{dI_{M}^{i}}{dt}=ac\left(\sum_{j} \omega_{ij}\left(I_{0}^{j}+I_{L}^{j}\right)\right)\left(I_{0}^{i}+I_{L}^{i}\right)\left(e^{-gn}-I_{M}^{i}\right)-gI_{M}^{i} \end{matrix}$$

The model for temperate strains of *P. vivax* with an exponentially distributed long-dormancy period described in equation (S15) can also be extended to account for multiple strains:

$$\begin{matrix} \frac{dS_{0}^{i}}{dt}=-\lambda^{i}S_{0}^{i}+rI_{0}^{i}+\gamma_{L}^{i}S_{L}^{i}+\gamma_{D}^{i}S_{D}^{i} \\ \frac{dI_{0}^{i}}{dt}=-\lambda^{i}I_{0}^{i}-rI_{0}^{i}+\gamma_{L}^{i}I_{L}^{i}+\gamma_{D}^{i}I_{D}^{i} \\ \frac{dS_{L}^{i}}{dt}=-\lambda^{i}S_{L}^{i}-f^{i}S_{L}^{i}e^{-\sum_{j} \psi_{ij}\left(I_{0}^{j}+I_{L}^{j}+I_{D}^{j}+I_{LD}^{j}\right)}+rI_{L}^{i}-\gamma_{L}^{i}S_{L}^{i}+\gamma_{D}^{i}S_{LD}^{i}+\delta^{i}S_{D}^{i}+\delta^{i}S_{LD}^{i} \\ \frac{dI_{L}^{i}}{dt}=f^{i}S_{L}^{i}e^{-\sum_{j} \psi_{ij}\left(I_{0}^{j}+I_{L}^{j}+I_{D}^{j}+I_{LD}^{j}\right)}-\lambda^{i}I_{L}^{i}-rI_{L}^{i}-\gamma_{L}^{i}I_{L}^{i}+\gamma_{D}^{i}I_{LD}^{i}+\delta^{i}I_{D}^{i}+\delta^{i}I_{LD}^{i} \\ \frac{dS_{D}^{i}}{dt}=\lambda^{i}S_{0}^{i}\left(1-e^{-\sum_{j} \psi_{ij}\left(I_{0}^{j}+I_{L}^{j}+I_{D}^{j}+I_{LD}^{j}\right)}\right)-\lambda^{i}S_{D}^{i}e^{-\sum_{j} \psi_{ij}\left(I_{0}^{j}+I_{L}^{j}+I_{D}^{j}+I_{LD}^{j}\right)}+rI_{D}^{i}+\gamma_{L}^{i}S_{LD}^{i}-\gamma_{D}^{i}S_{D}^{i}-\delta^{i}S_{D}^{i} \\ \frac{dI_{D}^{i}}{dt}=\left(\lambda^{i}S_{0}^{i}+\lambda^{i}S_{D}^{i}\right)e^{-\sum_{j} \psi_{ij}\left(I_{0}^{j}+I_{L}^{j}+I_{D}^{j}+I_{LD}^{j}\right)}+\lambda^{i}I_{0}^{i}-rI_{D}^{i}+\gamma_{L}^{i}I_{LD}^{i}-\gamma_{D}^{i}I_{D}^{i}-\delta^{i}I_{D}^{i} \\ \frac{dS_{LD}^{i}}{dt}=\lambda^{i}S_{L}^{i}\left(1-e^{-\sum_{j} \psi_{ij}\left(I_{0}^{j}+I_{L}^{j}+I_{D}^{j}+I_{LD}^{j}\right)}\right)-\left(\lambda^{i}S_{LD}^{i}+f^{i}S_{LD}^{i}\right)e^{-\sum_{j} \psi_{ij}\left(I_{0}^{j}+I_{L}^{j}+I_{D}^{j}+I_{LD}^{j}\right)}+rI_{LD}^{i}-\gamma_{L}^{i}S_{LD}^{i}-\gamma_{D}^{i}S_{LD}^{i}-\delta^{i}S_{LD}^{i} \\ \frac{dI_{LD}^{i}}{dt}=\left(\lambda^{i}S_{L}^{i}+\lambda^{i}S_{LD}^{i}+f^{i}S_{LD}^{i}\right)e^{-\sum_{j} \psi_{ij}\left(I_{0}^{j}+I_{L}^{j}+I_{D}^{j}+I_{LD}^{j}\right)}+\lambda^{i}I_{L}^{i}-rI_{LD}^{i}-\gamma_{L}^{i}I_{LD}^{i}-\gamma_{D}^{i}I_{LD}^{i}-\delta^{i}I_{LD}^{i} \\ \frac{dS_{M}^{i}}{dt}=g-ac\left(\sum_{j} \omega_{ij}\left(I_{0}^{j}+I_{L}^{j}+I_{D}^{j}+I_{LD}^{j}\right)\right)\left(e^{-gn}-I_{M}^{i}\right)-gS_{M}^{i} \\ \frac{dI_{M}^{i}}{dt}=ac\left(\sum_{j} \omega_{ij}\left(I_{0}^{j}+I_{L}^{j}+I_{D}^{j}+I_{LD}^{j}\right)\right)\left(e^{-gn}-I_{M}^{i}\right)-gI_{M}^{i} \end{matrix}$$

The model for temperate strains of *P. vivax* with a Gamma distributed long-dormancy period described in equation (S17) can also be extended to account for multiple strains:

**References**

49. Therneau TM. A package for survival analysis in S. 2015.

50. Rabarijaona LP, Randrianarivelojosia M, Raharimalala LA, Ratsimbasoa A, Randriamanantena A, Randrianasolo L, *et al*. Longitudinal survey of malaria morbidity over 10 years in Saharevo (Madagascar): further lessons for strengthening malaria control. Malar J. 2009; 8

51. Breeland SG. Methods for measuring *Anopheline* densities in El-Salvador. Mosquito News. 1972; 32(1):62-&

52. Herrel N, Amerasinghe FP, Ensink J, Mukhtar M, van der Hoek W, Konradsen F. Adult *Anopheline* ecology and malaria transmission in irrigated areas of South Punjab, Pakistan. Med Vet Entomol. 2004; 18:141-52.

53. van den Driessche P, Watmough J. Reproduction numbers and sub-threshold endemic equilibria for compartmental models of disease transmission. Math Biosci. 2002; 180:29-48

54. Griffin JT, Hollingsworth TD, Okell LC, Churcher TS, White M, Hinsley W, *et al*. Reducing *Plasmodium falciparum* malaria transmission in Africa: A model-based evaluation of intervention strategies. PLoS Med. 2010; 7(8)

55. McKenzie FE, Bossert WH. The optimal production of gametocytes by *Plasmodium falciparum*. J Theor Biol. 1998; 193(3):419-28

56. Kammanee A, Kanyamee N, Tang IM. Basic reproduction number for the transmission of *Plasmodium vivax* malaria. SE Asian J Trop Med Pub Health. 2001; 32(4):702-6

57. Aguas R, Ferreira MU, Gomes MGM. Modeling the effects of relapse in the transmission dynamics of malaria parasites. J Parasitol Res. 2012:921715

58. Pongsumpun P, Tang IM. Transmission model for *Plasmodium vivax* malaria: conditions for bifurcation. Int J Biol, Food, Vet Ag Engineer. 2007; 1(5).

59. Pongsumpun P, Mumtong P. Mathematical model for the incubation of the *Plasmodium vivax* malaria. Int J Appl Biomed Engineer. 2011; 4(1).
